# (NH_4_)Ga(HAsO_4_)_2_ and TlAl(HAsO_4_)_2_ - two new RbFe(HPO_4_)_2_-type *M* ^+^ *M* ^3+^ arsenates

**DOI:** 10.1107/S2056989018013567

**Published:** 2018-09-28

**Authors:** Karolina Schwendtner, Uwe Kolitsch

**Affiliations:** a Institute for Chemical Technology and Analytics, Division of Structural Chemistry, TU Wien, Getreidemarkt 9/164-SC, 1060 Vienna, Austria; b Naturhistorisches Museum, Burgring 7, 1010 Wien, and Institut für Mineralogie und Kristallographie, Universität Wien, Althanstrasse 14, 1090 Wien, Austria

**Keywords:** crystal structure, (NH_4_)Ga(HAsO_4_)_2_, TlAl(HAsO_4_)_2_

## Abstract

The crystal structures of hydro­thermally synthesized (*T* = 493 K, 7–9 d) ammonium gallium bis­[hydrogen arsenate(V)], (NH_4_)Ga(HAsO_4_)_2_, and thallium aluminium bis­[hydrogen arsenate(V)], TlAl(HAsO_4_)_2_, were solved by single-crystal X-ray diffraction. Both compounds crystallize in the common RbFe(HPO_4_)_2_ structure type (*R*  *c*) and share the same tetra­hedral–octa­hedral framework topology that houses the *M* ^+^ cations in its channels. One of the two Tl sites is slightly offset from its ideal position. Strong O—H⋯O hydrogen bonds strengthen the network.

## Abstract 2

The crystal structures of hydro­thermally synthesized (NH_4_)Ga(HAsO_4_)_2_ and TlAl(HAsO_4_)_2_ were solved by single-crystal X-ray diffraction. They both crystallize in the common RbFe(HPO_4_)_2_ structure type (*R* 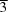 *c*).

## Chemical context   

Compounds with mixed tetra­hedral–octa­hedral (T–O) framework structures feature a broad range of different atomic arrangements. These result in topologies with several inter­esting properties such as ion exchange (Masquelier *et al.*, 1996 ▸) and ion conductivity (Chouchene *et al.*, 2017 ▸), as well as unusual piezoelectric (Ren *et al.*, 2015 ▸), magnetic (Ouerfelli *et al.*, 2007 ▸) or non-linear optical features (frequency doubling) (Sun *et al.*, 2017 ▸).

The two new compounds were obtained during an extensive experimental study of the system *M* ^+^–*M* ^3+^–O–(H)–As^5+^ (*M* ^+^ = Li, Na, K, Rb, Cs, Ag, Tl, NH_4_; *M* ^3+^ = Al, Ga, In, Sc, Fe, Cr, Tl), which led to an unusually large variety of new structure types (Schwendtner & Kolitsch, 2004 ▸, 2005 ▸, 2007*a* ▸,*b* ▸,*c* ▸, 2017*a* ▸, 2018*a* ▸; Schwendtner, 2006 ▸, 2008 ▸). Among the many different structure types found during our study, one atomic arrangement, the RbFe(HPO_4_)_2_ type (Lii & Wu, 1994 ▸; rhombohedral, *R* 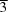 *c*), was found to exhibit a large crystal–chemical flexibility, which allows the incorporation of a wide variety of *M* ^+^ and *M* ^3+^ cations. Previously, it was also known for the phosphate members RbAl(HPO_4_)_2_ and RbGa(HPO_4_)_2_ (Lesage *et al.*, 2007 ▸). Currently (including the present paper), a total of eight arsenate members are known with the following *M* ^+^ *M* ^3+^ combinations: TlAl and (NH_4_)Ga (this work), RbIn, RbGa, RbAl, RbFe, CsIn and CsFe (Schwendtner & Kolitsch, 2017*b* ▸, 2018*a* ▸,*b* ▸,*c* ▸). It is noteworthy that no K members are currently known.

## Structural commentary   

**Figure 1:**
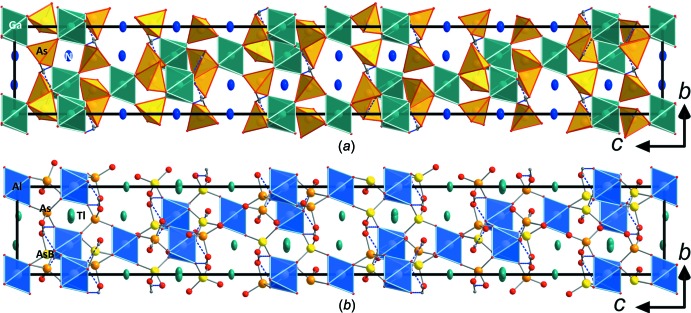
Structure drawings of the framework structures of (*a*) (NH_4_)Ga(HAsO_4_)_2_ and (*b*) TlAl(HAsO_4_)_2_ viewed along *a*. The unit cell is outlined and the alternative position As*B* in (*b*) is shown in light yellow (the main As position is orange). The Tl1 atom shows a slight positional disorder and is slightly offset from the ideal position.

**Figure 2:**
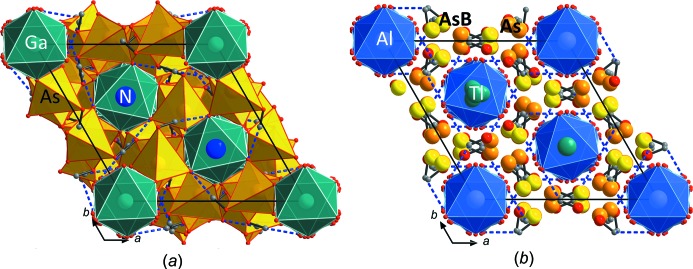
Structure drawings of the framework structures of (*a*) (NH_4_)Ga(HAsO_4_)_2_ and (*b*) TlAl(HAsO_4_)_2_ viewed along *c*. The unit cells are outlined and the alternative position As*B* in (*b*), which can be generated by a mirror plane in (110), is shown in light yellow (the main As position is orange). The Tl1 atom shows a slight positional disorder.

**Figure 3:**
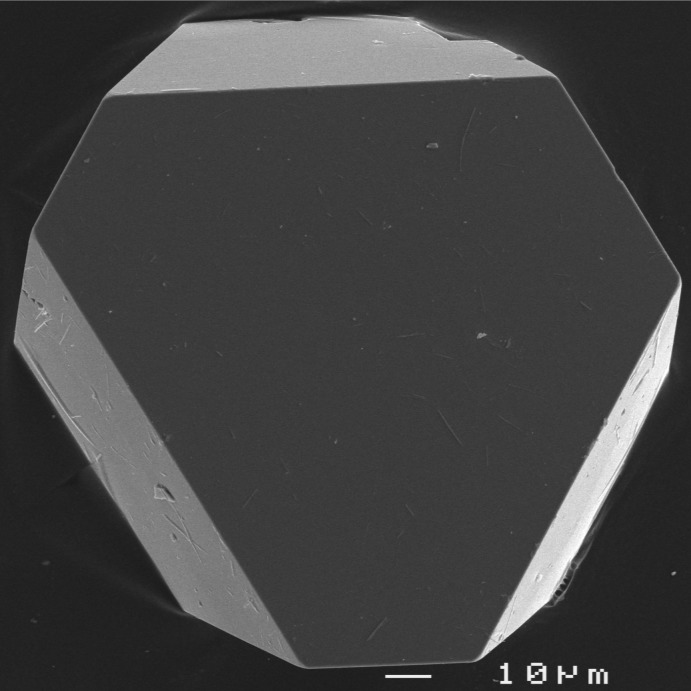
SEM image showing a flattened pseudo-octa­hedral crystal of (NH_4_)Ga(HAsO_4_)_2_.

The two compounds are representatives of the RbFe(HPO_4_)_2_ structure type (*R* 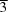 *c*; Lii & Wu, 1994 ▸) and show a basic tetra­hedral–octa­hedral framework structure featuring inter­penetrating channels, which host the *M* ^+^ cations (Fig. 1 ▸). This structure type is closely related to the triclinic (NH_4_)Fe(HPO_4_)_2_ type (*P* 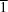; Yakubovich, 1993 ▸) in which all other known (NH_4_)*M^3^* ^+^(H*T*O_4_)_2_ (*T* = P, As) compounds crystallize (see Schwendtner & Kolitsch, 2018*b* ▸ for a compilation), the RbAl_2_As(HAsO_4_)_6_ type (*R* 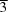 *c*; Schwendtner & Kolitsch, 2018*a* ▸) and the RbAl(HAsO_4_)_2_ type (*R*32; Schwendtner & Kolitsch, 2018*a* ▸). The fundamental building unit in all these structure types contains *M* ^3+^O_6_ octa­hedra, which are connected *via* their six corners to six protonated AsO_4_ tetra­hedra, thereby forming an *M* ^3+^As_6_O_24_ unit. These units are in turn connected *via* three corners to other *M* ^3+^O_6_ octa­hedra. The free, protonated corner of each AsO_4_ tetra­hedron forms a hydrogen bond to the neighbouring *M* ^3+^As_6_O_24_ group (Fig. 2 ▸). The *M* ^3+^As_6_O_24_ units are arranged in layers perpendicular to the *c*_hex_ axis (Fig. 1 ▸). The units within these layers are held together by medium–strong hydrogen bonds (Tables 1 ▸ and 2 ▸). Both title compounds invariably show a very similar crystal habit: strongly pseudo-hexa­gonal to pseudo-octa­hedral (*cf.* Fig. 3 ▸).

TlAl(HAsO_4_)_2_ has the smallest unit cell of all the arsenates of this structure type published to date. Still, the size of the *M* ^+^-hosting voids seems to be too large for the Tl^+^ cation, since Tl1 is slightly offset from the ideal position at 0, 0, 3/4 [resulting in some positional disorder for Tl1, with three symmetry-equivalent Tl1 positions in close proximity; Tl1–Tl1^i,ii^ = 0.28 (3) Å; symmetry codes: (i) −*y*, *x* − *y*, *z*; (ii) *y* − *x*, −*x*, *z*] and there are minor, but distinct negative and positive residual electron densities close to the Tl2 atom. The latter is severely underbonded, with a very low bond-valence sum (BVS) of only 0.54 valence units (v.u.) (calculated after Gagné & Hawthorne, 2015 ▸). The average Tl2—O bond length (Table 3 ▸) of 3.321 Å is considerably larger than the longest average Tl—O bond length of 3.304 Å described in the latest review paper (Gagné & Hawthorne, 2018 ▸), but still shorter than the excessively long average Tl—O bond length found in the related compound TlGa_2_As(HAsO_4_)_6_ (3.439 Å, Schwendtner & Kolitsch, 2018*b* ▸). The electron-density distribution is well fitted for the Tl1 atom, which has a BVS of 0.74 v.u. and an average Tl1—O bond length of 3.261 Å, which is also significantly longer than the reported average of 3.195 Å (Gagné & Hawthorne, 2018 ▸). In contrast, the two Al atoms are considerably overbonded (3.05 and 3.14 v.u. for Al1 and Al2, respectively) and average Al—O bond lengths of 1.898 and 1.887 Å are slightly shorter than the reported average of 1.903 Å (Gagné & Hawthorne, 2018 ▸), but well within the general range of Al—O bond lengths. The protonated AsO_4_ group shows a fairly typical configuration with slightly above average As—O bond lengths and a BVS of 4.97 v.u. for the As atom. As expected from the strong hydrogen bond [2.584 (5) Å, Table 2 ▸] the As—O bond to the donor O3 atom is considerably elongated (Table 3 ▸).

For (NH_4_)Ga(HAsO_4_)_2_, the bond-valence sum values for the *M* ^3+^ cations and As are quite similar (Table 4 ▸), with overbonded Ga^3+^ (BVS 3.10 and 3.15 v.u., respectively) and numbers for As that are close to the expected values (BVS 5.03 v.u., average bond length of 1.686 Å). The NH_4_^+^ cations (average N⋯O = 3.268 Å for N1 and 3.336 Å for N2) seem to fill the *M* ^+^-hosting voids much better, and the BVSs (calculated after García-Rodríguez *et al.*, 2000 ▸) of 0.74 and 1.03 v.u. for N1 and N2, respectively, are closer to ideal values, although N1 is underbonded.

## Synthesis and crystallization   

The compounds were grown by hydro­thermal synthesis at 493 K (autogeneous pressure, slow furnace cooling) using Teflon-lined stainless steel autoclaves with an approximate filling volume of 2 cm^3^. Reagent-grade NH_4_OH, Tl_2_CO_3_, Ga_2_O_3_, Al_2_O_3_ and H_3_AsO_4_·0.5H_2_O were used as starting reagents in approximate volume ratios of *M* ^+^:*M* ^3+^:As of 1:1:3 of the respective *M* ^+^ *M* ^3+^ compound for both synthesis batches. For TlAl(HAsO_4_)_2_, the vessels were filled with distilled water to about 70% of their inner volumes, which led to initial and final pH values of 1 and 0.5, respectively, and the synthesis was allowed to proceed at 493 K for 9 d. (NH_4_)Ga(HAsO_4_)_2_ was grown over a period of 7 d and the initial and final pH values were 3 and 1, respectively. The reaction products were washed thoroughly with distilled water, filtered, and dried at room temperature. (NH_4_)Ga(HAsO_4_)_2_ formed large colourless pseudo-octa­hedral crystals (Fig. 3 ▸), while TlAl(HAsO_4_)_2_ formed small pseudo-hexa­gonal platelets. Both compounds are stable in air.

A measured X-ray powder diffraction pattern of (NH_4_)Ga(HAsO_4_)_2_ was deposited at the Inter­national Centre for Diffraction Data under PDF number 00-059-0055 (Wohlschlaeger *et al.*, 2007 ▸).

Semiqu­anti­tative SEM–EDX analysis (15 kV) of carbon-coated, horizontally oriented crystals of (NH_4_)Ga(HAsO_4_)_2_ were undertaken to discriminate between H_3_O^+^ and NH_4_^+^. They confirmed the suspected formula and revealed no impurities.

## Refinement   

Crystal data, data collection, and structure refinement details are summarized in Table 5 ▸.

For the refinement of both compounds, the coordinates of RbFe(HPO_4_)_2_ (Lii & Wu, 1994 ▸) were used for the initial refinement steps. The hydrogen atoms were then located in difference-Fourier maps and added to the models. In both compounds O—H bonds were restrained to 0.9 ± 0.04 Å. In (NH_4_)Ga(HAsO_4_)_2_, several electron-density peaks between 0.4 and 0.75 e Å^−3^ were recognizable that could be attributed to the H atoms of the NH_4_^+^ cation. These peaks are located at the following coordinates for the N1 atom: 0.0170, 0.1329, 0.7450; 0.0641, 0.0560, 0.7414 and −0.0910, 0.0000, 0.7500. For the N2 atom, the coordinates are: 0.0478, −0.0330, 0.6635; −0.0655, −0.1106, 0.6786; 0.1301, 0.0094, 0.6695 and −0.0521, −0.0657, 0.6513. However, despite the use of restraints, no sensible coordination geometry for the H atoms around the N atoms could be found. Therefore, they were omitted from the model. As a result of the fact that there are 12 possible N—H⋯O bonds for each N atom, with only two symmetry-equivalent positions for N1 and four for N2, it seems reasonable to assume that the H-atom positions around the N atoms are, in both cases, highly disordered. The final residual electron density in (NH_4_)Ga(HAsO_4_)_2_ is < 1e Å^−3^.

The refinement of TlAl(HAsO_4_)_2_ revealed a considerable residual electron-density peak of 2.2 e Å^−3^ 1.28 Å away from As and 1.61 Å away from the O1 site. The corresponding position can be generated by a mirror plane in (110) and therefore could be an alternative flipped As position (sharing the same O1 atom). Since the inclusion of the alternative position led to a considerable drop in *R*_1_ and weighting parameters and the highest residual electron density dropped to < 1 e Å^−3^, this position was kept in the model. The occupancy of the alternative position As*B* (Fig. 1 ▸ *b*, 2*b*) refined to only 2.1%, which makes it impossible to locate the alternative O ligand positions that should comprise the coordination sphere of the As*B* position. For the final refinement, the displacement parameters of the As*B* position were restrained to be the same as for the main As position and the sum of As was restrained to give a total occupancy of 1.00. We note that a similar alternative position was also found for isotypic CsIn(HAsO_4_)_2_ (Schwendtner & Kolitsch, 2017*b* ▸).

There was also considerable residual electron density of ±2 e Å ^−3^ close to the two Tl positions, similar to what was encountered in the structurally related TlGa_2_As(HAsO_4_)_6_ (Schwendtner & Kolitsch, 2018*d* ▸). We tried a similar approach that had worked well for the aforementioned compound, *viz*. to remove the Tl atoms from their ideal, highly symmetrical positions in this structure type. We obtained a better refinement with a slightly off-centre position for Tl1, in line with a slight disorder (probably static), possibly in part or in whole due to the stereochemical activity of the lone electron pair on the Tl^+^ cations. So, although the Tl1 site is slightly offset from its ideal position (0, 0, 3/4), we unfortunately did not manage to get rid of the negative residual electron density of about −2 e Å^−3^ next to Tl2. The most positive residual electron density peak, however, dropped to < 1 e Å^−3^.

## Supplementary Material

Crystal structure: contains datablock(s) NH4GaHAsO42, TlAlHAsO42. DOI: 10.1107/S2056989018013567/pk2608sup1.cif

Structure factors: contains datablock(s) NH4GaHAsO42. DOI: 10.1107/S2056989018013567/pk2608NH4GaHAsO42sup2.hkl

Structure factors: contains datablock(s) TlAlHAsO42. DOI: 10.1107/S2056989018013567/pk2608TlAlHAsO42sup3.hkl

CCDC references: 1869299, 1869298

Additional supporting information:  crystallographic information; 3D view; checkCIF report

## Figures and Tables

**Table 1:** Hydrogen-bond geometry (Å, °) for (NH_4_)Ga(HAsO_4_)

*D*—H⋯*A*	*D*—H	H⋯*A*	*D*⋯*A*	*D*—H⋯*A*
O3—H3⋯O4^xxi^	0.87 (3)	1.74 (3)	2.610 (3)	172 (6)

Symmetry code: (xxi) 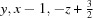.

**Table 2:** Hydrogen-bond geometry (Å, °) for TlAl(HAsO_4_)_2_

*D*—H⋯*A*	*D*—H	H⋯*A*	*D*⋯*A*	*D*—H⋯*A*
O3—H3⋯O4^xxi^	0.87 (4)	1.87 (5)	2.584 (5)	139 (6)

Symmetry code: (xxi) 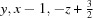.

**Table 3:** Selected bond lengths (Å) for TlAl(HAsO_4_)_2_

Tl1—Tl1^i^	0.28 (3)	Tl2—O4^xi^	3.516 (3)
Tl1—O3	3.085 (8)	Tl2—O3^xii^	3.545 (4)
Tl1—O3^ii^	3.085 (8)	Tl2—O3^xiii^	3.545 (4)
Tl1—O3^iii^	3.136 (5)	Tl2—O3^xiv^	3.545 (4)
Tl1—O3^i^	3.136 (5)	Al1—O2^xv^	1.895 (4)
Tl1—O2^iii^	3.233 (13)	Al1—O2^v^	1.895 (4)
Tl1—O2^i^	3.233 (13)	Al1—O2^xvi^	1.895 (4)
Tl1—O3^iv^	3.261 (12)	Al1—O4^xvii^	1.901 (4)
Tl1—O3^v^	3.261 (12)	Al1—O4^i^	1.901 (4)
Tl1—O2^ii^	3.351 (4)	Al1—O4^xviii^	1.901 (4)
Tl1—O2	3.351 (4)	Al2—O1^viii^	1.887 (4)
Tl1—O2^v^	3.501 (15)	Al2—O1^xiv^	1.887 (4)
Tl1—O2^iv^	3.501 (15)	Al2—O1^xix^	1.887 (4)
Tl2—O3^i^	2.813 (4)	Al2—O1^i^	1.887 (4)
Tl2—O3^v^	2.813 (4)	Al2—O1^xviii^	1.887 (4)
Tl2—O3	2.813 (4)	Al2—O1^xvii^	1.887 (4)
Tl2—O1^vi^	3.410 (4)	As—O1^xx^	1.661 (3)
Tl2—O1^vii^	3.410 (4)	As—O2	1.674 (3)
Tl2—O1^viii^	3.410 (4)	As—O4^ii^	1.679 (3)
Tl2—O4^ix^	3.516 (3)	As—O3	1.746 (4)
Tl2—O4^x^	3.516 (3)		

Symmetry codes: (i) 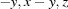; (ii) 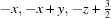; (iii) 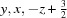; (iv) 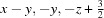; (v) 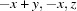; (vi) 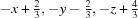; (vii) 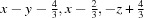; (viii) 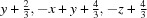; (ix) 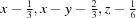; (x) 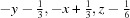; (xi) 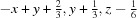; (xii) 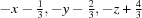; (xiii) 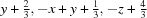; (xiv) 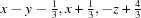; (xv) 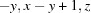; (xvi) 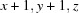; (xvii) 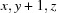; (xviii) 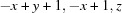; (xix) 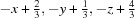; (xx) 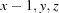.

**Table 4:** Selected bond lengths (Å) for (NH_4_)Ga(HAsO_4_)

N1—O3	3.173 (3)	N2—O4^xi^	3.493 (5)
N1—O3^i^	3.173 (3)	N2—O3^xii^	3.557 (4)
N1—O3^ii^	3.173 (3)	N2—O3^xiii^	3.557 (4)
N1—O3^iii^	3.173 (3)	N2—O3^xiv^	3.557 (4)
N1—O3^iv^	3.173 (3)	Ga1—O2^xv^	1.9619 (16)
N1—O3^v^	3.173 (3)	Ga1—O2^iii^	1.9619 (17)
N1—O2	3.3657 (18)	Ga1—O2^xvi^	1.9619 (17)
N1—O2^ii^	3.3657 (18)	Ga1—O4^v^	1.9666 (17)
N1—O2^iv^	3.3657 (18)	Ga1—O4^xvii^	1.9666 (17)
N1—O2^iii^	3.3657 (18)	Ga1—O4^xviii^	1.9667 (16)
N1—O2^i^	3.3657 (17)	Ga2—O1^viii^	1.9588 (18)
N1—O2^v^	3.3657 (17)	Ga2—O1^xiv^	1.9588 (19)
N2—O3^v^	2.918 (4)	Ga2—O1^xix^	1.9588 (18)
N2—O3^iii^	2.918 (4)	Ga2—O1^v^	1.9589 (18)
N2—O3	2.918 (4)	Ga2—O1^xviii^	1.9589 (19)
N2—O1^vi^	3.375 (3)	Ga2—O1^xvii^	1.9589 (18)
N2—O1^vii^	3.375 (3)	As—O1^xx^	1.6555 (18)
N2—O1^viii^	3.375 (3)	As—O2	1.6700 (16)
N2—O4^ix^	3.493 (5)	As—O4^ii^	1.6783 (17)
N2—O4^x^	3.493 (5)	As—O3	1.740 (2)

Symmetry codes: (i) 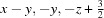; (ii) 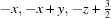; (iii) 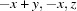; (iv) 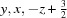; (v) 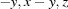; (vi) 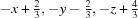; (vii) 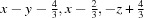; (viii) 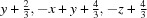; (ix) 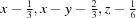; (x) 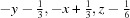; (xi) 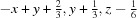; (xii) 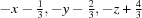; (xiii) 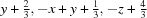; (xiv) 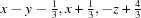; (xv) 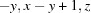; (xvi) 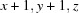; (xvii) 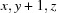; (xviii) 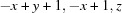; (xix) 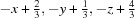; (xx) 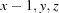.

**Table 5:** Experimental details

	(NH_4_)Ga(HAsO_4_)_2_	TlAl(HAsO_4_)_2_
Crystal data		
*M*_r_	367.62	511.21
Crystal system, space group	Trigonal, *R* 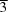 *c*:*H*	Trigonal, *R* 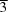 *c*:*H*
Temperature (K)	293	293
*a*, *c* (Å)	8.380 (1), 53.811 (11)	8.290 (1), 52.940 (11)
*V* (Å^3^)	3272.6 (10)	3150.8 (10)
*Z*	18	18
Radiation type	Mo *K*α	Mo *K*α
μ (mm^−1^)	12.83	32.58
Crystal size (mm)	0.08 × 0.07 × 0.03	0.08 × 0.07 × 0.03

Data collection		
Diffractometer	Nonius KappaCCD single-crystal four-circle diffractometer	Nonius KappaCCD single-crystal four-circle
Absorption correction	Multi-scan (*HKL* *SCALEPACK*; Otwinowski *et al.*, 2003 ▸)	Multi-scan (*HKL* *SCALEPACK*; Otwinowski *et al.*, 2003 ▸)
*T*_min_, *T*_max_	0.427, 0.700	0.180, 0.441
No. of measured, independent and observed [*I* > 2σ(*I*)] reflections	4834, 1326, 1156	2478, 698, 685
*R*_int_	0.024	0.022
(sin θ/λ)_max_ (Å^−1^)	0.757	0.617

Refinement		
*R*[*F* ^2^ > 2σ(*F* ^2^)], *wR*(*F* ^2^), *S*	0.022, 0.055, 1.07	0.022, 0.058, 1.21
No. of reflections	1326	698
No. of parameters	61	69
No. of restraints	1	2
H-atom treatment	All H-atom parameters refined	All H-atom parameters refined
Δρ_max_, Δρ_min_ (e Å^−3^)	0.75, −0.95	0.82, −1.98

Computer programs: *COLLECT* (Nonius, 2003 ▸), *HKL* *DENZO* and *SCALEPACK* (Otwinowski *et al.*, 2003 ▸), *SHELXS97* (Sheldrick, 2008 ▸), *SHELXL2016* (Sheldrick, 2015 ▸), *DIAMOND* (Brandenburg, 2005 ▸) and *publCIF* (Westrip, 2010 ▸).

## supplementary crystallographic information

### Ammonium gallium bis[hydrogen arsenate(V)] (NH4GaHAsO42) Crystal data

**Table:**

(NH_4_)Ga(HAsO_4_)_2_	*D*_x_ = 3.358 Mg m^−^^3^
*M**_r_* = 367.62	Mo *K*α radiation, λ = 0.71073 Å
Trigonal, *R*3*c*:*H*	Cell parameters from 2653 reflections
*a* = 8.380 (1) Å	θ = 2.9–32.5°
*c* = 53.811 (11) Å	µ = 12.83 mm^−^^1^
*V* = 3272.6 (10) Å^3^	*T* = 293 K
*Z* = 18	Small pseudo-octahedral platelets, colourless
*F*(000) = 3132	0.08 × 0.07 × 0.03 mm

### Ammonium gallium bis[hydrogen arsenate(V)] (NH4GaHAsO42) Data collection

**Table:**

Nonius KappaCCD single-crystal four-circle diffractometer	1156 reflections with *I* > 2σ(*I*)
Radiation source: fine-focus sealed tube	*R*_int_ = 0.024
φ and ω scans	θ_max_ = 32.5°, θ_min_ = 2.9°
Absorption correction: multi-scan (HKL SCALEPACK; Otwinowski *et al.*, 2003)	*h* = −12→12
*T*_min_ = 0.427, *T*_max_ = 0.700	*k* = −10→10
4834 measured reflections	*l* = −80→81
1326 independent reflections	

### Ammonium gallium bis[hydrogen arsenate(V)] (NH4GaHAsO42) Refinement

**Table:**

Refinement on *F*^2^	Secondary atom site location: difference Fourier map
Least-squares matrix: full	Hydrogen site location: difference Fourier map
*R*[*F*^2^ > 2σ(*F*^2^)] = 0.022	All H-atom parameters refined
*wR*(*F*^2^) = 0.055	*w* = 1/[σ^2^(*F*_o_^2^) + (0.0273*P*)^2^ + 16.8283*P*] where *P* = (*F*_o_^2^ + 2*F*_c_^2^)/3
*S* = 1.07	(Δ/σ)_max_ = 0.003
1326 reflections	Δρ_max_ = 0.75 e Å^−^^3^
61 parameters	Δρ_min_ = −0.95 e Å^−^^3^
1 restraint	Extinction correction: SHELXL2016 (Sheldrick, 2015), Fc^*^=kFc[1+0.001xFc^2^λ^3^/sin(2θ)]^-1/4^
Primary atom site location: structure-invariant direct methods	Extinction coefficient: 0.00016 (3)

### Ammonium gallium bis[hydrogen arsenate(V)] (NH4GaHAsO42) Special details

**Table:**

Geometry. All esds (except the esd in the dihedral angle between two l.s. planes) are estimated using the full covariance matrix. The cell esds are taken into account individually in the estimation of esds in distances, angles and torsion angles; correlations between esds in cell parameters are only used when they are defined by crystal symmetry. An approximate (isotropic) treatment of cell esds is used for estimating esds involving l.s. planes.

### Ammonium gallium bis[hydrogen arsenate(V)] (NH4GaHAsO42) Fractional atomic coordinates and isotropic or equivalent isotropic displacement parameters (Å^2^)

**Table:**

	*x*	*y*	*z*	*U*_iso_*/*U*_eq_	
N1	0.000000	0.000000	0.750000	0.051 (2)	
N2	0.000000	0.000000	0.66731 (10)	0.0487 (15)	
Ga1	0.333333	0.666667	0.75382 (2)	0.00954 (10)	
Ga2	0.333333	0.666667	0.666667	0.01164 (13)	
As	−0.42915 (3)	−0.39386 (3)	0.71282 (2)	0.01072 (8)	
O1	0.4557 (3)	−0.4378 (3)	0.68635 (3)	0.0218 (4)	
O2	−0.4457 (2)	−0.2535 (2)	0.73337 (3)	0.0133 (3)	
O3	−0.1958 (3)	−0.2785 (3)	0.70541 (4)	0.0243 (4)	
O4	0.4778 (2)	−0.1224 (2)	0.77594 (3)	0.0127 (3)	
H3	−0.161 (8)	−0.353 (6)	0.7114 (9)	0.075 (18)*	

### Ammonium gallium bis[hydrogen arsenate(V)] (NH4GaHAsO42) Atomic displacement parameters (Å^2^)

**Table:**

	*U*^11^	*U*^22^	*U*^33^	*U*^12^	*U*^13^	*U*^23^
N1	0.062 (4)	0.062 (4)	0.029 (4)	0.0311 (18)	0.000	0.000
N2	0.060 (2)	0.060 (2)	0.026 (2)	0.0300 (12)	0.000	0.000
Ga1	0.01025 (13)	0.01025 (13)	0.00811 (19)	0.00513 (6)	0.000	0.000
Ga2	0.01394 (18)	0.01394 (18)	0.0070 (2)	0.00697 (9)	0.000	0.000
As	0.01365 (12)	0.01158 (12)	0.00927 (12)	0.00807 (9)	0.00172 (8)	0.00141 (7)
O1	0.0368 (11)	0.0281 (10)	0.0101 (7)	0.0234 (9)	−0.0049 (7)	−0.0010 (7)
O2	0.0137 (7)	0.0121 (7)	0.0135 (7)	0.0061 (6)	0.0030 (6)	−0.0014 (6)
O3	0.0192 (9)	0.0220 (9)	0.0362 (12)	0.0137 (8)	0.0137 (8)	0.0126 (8)
O4	0.0136 (7)	0.0108 (7)	0.0153 (7)	0.0073 (6)	−0.0026 (6)	−0.0047 (6)

### Ammonium gallium bis[hydrogen arsenate(V)] (NH4GaHAsO42) Geometric parameters (Å, º)

**Table:**

N1—O3	3.173 (3)	N2—O3^xii^	3.557 (4)
N1—O3^i^	3.173 (3)	N2—O3^xiii^	3.557 (4)
N1—O3^ii^	3.173 (3)	N2—O3^xiv^	3.557 (4)
N1—O3^iii^	3.173 (3)	Ga1—O2^xv^	1.9619 (16)
N1—O3^iv^	3.173 (3)	Ga1—O2^iii^	1.9619 (17)
N1—O3^v^	3.173 (3)	Ga1—O2^xvi^	1.9619 (17)
N1—O2	3.3657 (18)	Ga1—O4^v^	1.9666 (17)
N1—O2^ii^	3.3657 (18)	Ga1—O4^xvii^	1.9666 (17)
N1—O2^iv^	3.3657 (18)	Ga1—O4^xviii^	1.9667 (16)
N1—O2^iii^	3.3657 (18)	Ga2—O1^viii^	1.9588 (18)
N1—O2^i^	3.3657 (17)	Ga2—O1^xiv^	1.9588 (19)
N1—O2^v^	3.3657 (17)	Ga2—O1^xix^	1.9588 (18)
N2—O3^v^	2.918 (4)	Ga2—O1^v^	1.9589 (18)
N2—O3^iii^	2.918 (4)	Ga2—O1^xviii^	1.9589 (19)
N2—O3	2.918 (4)	Ga2—O1^xvii^	1.9589 (18)
N2—O1^vi^	3.375 (3)	As—O1^xx^	1.6555 (18)
N2—O1^vii^	3.375 (3)	As—O2	1.6700 (16)
N2—O1^viii^	3.375 (3)	As—O4^ii^	1.6783 (17)
N2—O4^ix^	3.493 (5)	As—O3	1.740 (2)
N2—O4^x^	3.493 (5)	O3—H3	0.87 (3)
N2—O4^xi^	3.493 (5)		
			
O3—N1—O3^i^	162.83 (7)	O2^xv^—Ga1—N1	119.85 (5)
O3—N1—O3^ii^	123.01 (7)	O2^iii^—Ga1—N1	32.80 (5)
O3^i^—N1—O3^ii^	69.03 (6)	O2^xvi^—Ga1—N1	105.75 (5)
O3—N1—O3^iii^	69.03 (6)	O4^v^—Ga1—N1	77.33 (5)
O3^i^—N1—O3^iii^	102.44 (7)	O4^xvii^—Ga1—N1	143.46 (5)
O3^ii^—N1—O3^iii^	162.83 (8)	O4^xviii^—Ga1—N1	59.54 (5)
O3—N1—O3^iv^	102.44 (7)	N2^xxi^—Ga1—N1	92.432 (5)
O3^i^—N1—O3^iv^	69.03 (6)	N1^xvii^—Ga1—N1	119.821 (1)
O3^ii^—N1—O3^iv^	69.03 (6)	O2^xv^—Ga1—N1^xvi^	105.75 (5)
O3^iii^—N1—O3^iv^	123.01 (7)	O2^iii^—Ga1—N1^xvi^	119.85 (5)
O3—N1—O3^v^	69.03 (6)	O2^xvi^—Ga1—N1^xvi^	32.80 (5)
O3^i^—N1—O3^v^	123.01 (8)	O4^v^—Ga1—N1^xvi^	143.46 (5)
O3^ii^—N1—O3^v^	102.44 (7)	O4^xvii^—Ga1—N1^xvi^	59.54 (5)
O3^iii^—N1—O3^v^	69.03 (6)	O4^xviii^—Ga1—N1^xvi^	77.33 (5)
O3^iv^—N1—O3^v^	162.83 (7)	N2^xxi^—Ga1—N1^xvi^	92.432 (5)
O3—N1—O2	48.11 (4)	N1^xvii^—Ga1—N1^xvi^	119.821 (1)
O3^i^—N1—O2	115.51 (5)	N1—Ga1—N1^xvi^	119.821 (1)
O3^ii^—N1—O2	126.51 (5)	O1^viii^—Ga2—O1^xiv^	93.53 (7)
O3^iii^—N1—O2	70.41 (5)	O1^viii^—Ga2—O1^xix^	93.53 (7)
O3^iv^—N1—O2	65.01 (5)	O1^xiv^—Ga2—O1^xix^	93.53 (7)
O3^v^—N1—O2	113.56 (5)	O1^viii^—Ga2—O1^v^	180.0
O3—N1—O2^ii^	126.52 (5)	O1^xiv^—Ga2—O1^v^	86.47 (7)
O3^i^—N1—O2^ii^	70.41 (5)	O1^xix^—Ga2—O1^v^	86.47 (7)
O3^ii^—N1—O2^ii^	48.11 (5)	O1^viii^—Ga2—O1^xviii^	86.47 (7)
O3^iii^—N1—O2^ii^	115.51 (5)	O1^xiv^—Ga2—O1^xviii^	180.0
O3^iv^—N1—O2^ii^	113.56 (5)	O1^xix^—Ga2—O1^xviii^	86.47 (7)
O3^v^—N1—O2^ii^	65.01 (5)	O1^v^—Ga2—O1^xviii^	93.53 (7)
O2—N1—O2^ii^	171.26 (6)	O1^viii^—Ga2—O1^xvii^	86.47 (7)
O3—N1—O2^iv^	65.01 (5)	O1^xiv^—Ga2—O1^xvii^	86.47 (7)
O3^i^—N1—O2^iv^	113.56 (5)	O1^xix^—Ga2—O1^xvii^	180.0
O3^ii^—N1—O2^iv^	70.41 (5)	O1^v^—Ga2—O1^xvii^	93.53 (7)
O3^iii^—N1—O2^iv^	126.51 (5)	O1^xviii^—Ga2—O1^xvii^	93.53 (7)
O3^iv^—N1—O2^iv^	48.11 (4)	O1^viii^—Ga2—N2^xix^	62.79 (8)
O3^v^—N1—O2^iv^	115.51 (5)	O1^xiv^—Ga2—N2^xix^	67.00 (7)
O2—N1—O2^iv^	59.00 (6)	O1^xix^—Ga2—N2^xix^	146.77 (8)
O2^ii^—N1—O2^iv^	113.20 (2)	O1^v^—Ga2—N2^xix^	117.21 (8)
O3—N1—O2^iii^	113.56 (5)	O1^xviii^—Ga2—N2^xix^	113.00 (7)
O3^i^—N1—O2^iii^	65.01 (5)	O1^xvii^—Ga2—N2^xix^	33.23 (8)
O3^ii^—N1—O2^iii^	115.51 (5)	O1^viii^—Ga2—N2^xvii^	117.21 (8)
O3^iii^—N1—O2^iii^	48.11 (5)	O1^xiv^—Ga2—N2^xvii^	113.00 (7)
O3^iv^—N1—O2^iii^	126.51 (5)	O1^xix^—Ga2—N2^xvii^	33.23 (8)
O3^v^—N1—O2^iii^	70.41 (5)	O1^v^—Ga2—N2^xvii^	62.79 (8)
O2—N1—O2^iii^	113.20 (2)	O1^xviii^—Ga2—N2^xvii^	66.99 (7)
O2^ii^—N1—O2^iii^	74.86 (6)	O1^xvii^—Ga2—N2^xvii^	146.77 (8)
O2^iv^—N1—O2^iii^	171.26 (6)	N2^xix^—Ga2—N2^xvii^	180.0
O3—N1—O2^i^	115.51 (5)	O1^viii^—Ga2—N2	33.23 (8)
O3^i^—N1—O2^i^	48.11 (4)	O1^xiv^—Ga2—N2	117.21 (8)
O3^ii^—N1—O2^i^	113.56 (5)	O1^xix^—Ga2—N2	113.01 (8)
O3^iii^—N1—O2^i^	65.01 (5)	O1^v^—Ga2—N2	146.77 (8)
O3^iv^—N1—O2^i^	70.41 (5)	O1^xviii^—Ga2—N2	62.79 (8)
O3^v^—N1—O2^i^	126.51 (5)	O1^xvii^—Ga2—N2	67.00 (8)
O2—N1—O2^i^	74.86 (6)	N2^xix^—Ga2—N2	60.005 (2)
O2^ii^—N1—O2^i^	113.20 (2)	N2^xvii^—Ga2—N2	119.995 (2)
O2^iv^—N1—O2^i^	113.20 (2)	O1^viii^—Ga2—N2^xvi^	113.01 (7)
O2^iii^—N1—O2^i^	59.00 (6)	O1^xiv^—Ga2—N2^xvi^	33.23 (8)
O3—N1—O2^v^	70.41 (5)	O1^xix^—Ga2—N2^xvi^	117.21 (8)
O3^i^—N1—O2^v^	126.51 (5)	O1^v^—Ga2—N2^xvi^	66.99 (7)
O3^ii^—N1—O2^v^	65.01 (5)	O1^xviii^—Ga2—N2^xvi^	146.77 (8)
O3^iii^—N1—O2^v^	113.56 (5)	O1^xvii^—Ga2—N2^xvi^	62.79 (8)
O3^iv^—N1—O2^v^	115.51 (5)	N2^xix^—Ga2—N2^xvi^	60.005 (2)
O3^v^—N1—O2^v^	48.11 (4)	N2^xvii^—Ga2—N2^xvi^	119.995 (2)
O2—N1—O2^v^	113.20 (2)	N2—Ga2—N2^xvi^	119.995 (2)
O2^ii^—N1—O2^v^	59.00 (6)	O1^viii^—Ga2—N2^xxii^	66.99 (7)
O2^iv^—N1—O2^v^	74.86 (6)	O1^xiv^—Ga2—N2^xxii^	146.77 (8)
O2^iii^—N1—O2^v^	113.20 (2)	O1^xix^—Ga2—N2^xxii^	62.79 (8)
O2^i^—N1—O2^v^	171.26 (6)	O1^v^—Ga2—N2^xxii^	113.00 (7)
O3^v^—N2—O3^iii^	76.09 (13)	O1^xviii^—Ga2—N2^xxii^	33.23 (8)
O3^v^—N2—O3	76.08 (13)	O1^xvii^—Ga2—N2^xxii^	117.21 (8)
O3^iii^—N2—O3	76.08 (13)	N2^xix^—Ga2—N2^xxii^	119.995 (2)
O3^v^—N2—O1^vi^	77.21 (6)	N2^xvii^—Ga2—N2^xxii^	60.005 (2)
O3^iii^—N2—O1^vi^	152.44 (16)	N2—Ga2—N2^xxii^	60.005 (2)
O3—N2—O1^vi^	91.02 (6)	N2^xvi^—Ga2—N2^xxii^	180.0
O3^v^—N2—O1^vii^	152.44 (16)	O1^viii^—Ga2—N2^xxiii^	146.77 (8)
O3^iii^—N2—O1^vii^	91.02 (6)	O1^xiv^—Ga2—N2^xxiii^	62.79 (8)
O3—N2—O1^vii^	77.21 (6)	O1^xix^—Ga2—N2^xxiii^	66.99 (8)
O1^vi^—N2—O1^vii^	110.03 (9)	O1^v^—Ga2—N2^xxiii^	33.23 (8)
O3^v^—N2—O1^viii^	91.02 (6)	O1^xviii^—Ga2—N2^xxiii^	117.21 (8)
O3^iii^—N2—O1^viii^	77.21 (6)	O1^xvii^—Ga2—N2^xxiii^	113.00 (8)
O3—N2—O1^viii^	152.44 (16)	N2^xix^—Ga2—N2^xxiii^	119.995 (2)
O1^vi^—N2—O1^viii^	110.03 (9)	N2^xvii^—Ga2—N2^xxiii^	60.005 (2)
O1^vii^—N2—O1^viii^	110.03 (9)	N2—Ga2—N2^xxiii^	180.0
O3^v^—N2—O4^ix^	111.48 (7)	N2^xvi^—Ga2—N2^xxiii^	60.005 (2)
O3^iii^—N2—O4^ix^	156.43 (10)	N2^xxii^—Ga2—N2^xxiii^	119.994 (2)
O3—N2—O4^ix^	126.99 (7)	O1^xx^—As—O2	118.81 (9)
O1^vi^—N2—O4^ix^	45.41 (6)	O1^xx^—As—O4^ii^	105.46 (9)
O1^vii^—N2—O4^ix^	90.09 (12)	O2—As—O4^ii^	115.11 (9)
O1^viii^—N2—O4^ix^	80.30 (10)	O1^xx^—As—O3	107.12 (11)
O3^v^—N2—O4^x^	156.43 (10)	O2—As—O3	103.09 (10)
O3^iii^—N2—O4^x^	126.99 (7)	O4^ii^—As—O3	106.35 (9)
O3—N2—O4^x^	111.48 (7)	O1^xx^—As—N2^xii^	64.22 (8)
O1^vi^—N2—O4^x^	80.30 (10)	O2—As—N2^xii^	173.25 (6)
O1^vii^—N2—O4^x^	45.41 (6)	O4^ii^—As—N2^xii^	68.25 (8)
O1^viii^—N2—O4^x^	90.09 (12)	O3—As—N2^xii^	70.17 (8)
O4^ix^—N2—O4^x^	45.67 (8)	O1^xx^—As—N1	142.98 (8)
O3^v^—N2—O4^xi^	126.99 (7)	O2—As—N1	56.21 (6)
O3^iii^—N2—O4^xi^	111.48 (7)	O4^ii^—As—N1	108.99 (6)
O3—N2—O4^xi^	156.43 (10)	O3—As—N1	50.09 (8)
O1^vi^—N2—O4^xi^	90.09 (12)	N2^xii^—As—N1	117.48 (2)
O1^vii^—N2—O4^xi^	80.30 (10)	O1^xx^—As—N2	81.32 (10)
O1^viii^—N2—O4^xi^	45.41 (6)	O2—As—N2	99.84 (7)
O4^ix^—N2—O4^xi^	45.67 (8)	O4^ii^—As—N2	133.25 (6)
O4^x^—N2—O4^xi^	45.67 (8)	O3—As—N2	32.26 (8)
O3^v^—N2—O3^xii^	119.40 (8)	N2^xii^—As—N2	74.310 (11)
O3^iii^—N2—O3^xii^	150.86 (8)	N1—As—N2	65.36 (6)
O3—N2—O3^xii^	83.78 (6)	O1^xx^—As—N1^xxiv^	88.02 (8)
O1^vi^—N2—O3^xii^	46.33 (6)	O2—As—N1^xxiv^	94.12 (6)
O1^vii^—N2—O3^xii^	63.78 (7)	O4^ii^—As—N1^xxiv^	40.77 (6)
O1^viii^—N2—O3^xii^	123.57 (16)	O3—As—N1^xxiv^	147.10 (7)
O4^ix^—N2—O3^xii^	45.67 (7)	N2^xii^—As—N1^xxiv^	92.00 (3)
O4^x^—N2—O3^xii^	43.44 (7)	N1—As—N1^xxiv^	127.434 (12)
O4^xi^—N2—O3^xii^	80.03 (12)	N2—As—N1^xxiv^	165.32 (5)
O3^v^—N2—O3^xiii^	83.77 (6)	O1^xx^—As—N2^xxiv^	43.28 (9)
O3^iii^—N2—O3^xiii^	119.40 (8)	O2—As—N2^xxiv^	127.15 (7)
O3—N2—O3^xiii^	150.86 (7)	O4^ii^—As—N2^xxiv^	63.82 (7)
O1^vi^—N2—O3^xiii^	63.78 (7)	O3—As—N2^xxiv^	128.79 (9)
O1^vii^—N2—O3^xiii^	123.57 (16)	N2^xii^—As—N2^xxiv^	59.42 (2)
O1^viii^—N2—O3^xiii^	46.33 (6)	N1—As—N2^xxiv^	172.65 (3)
O4^ix^—N2—O3^xiii^	43.44 (7)	N2—As—N2^xxiv^	117.94 (11)
O4^x^—N2—O3^xiii^	80.03 (12)	N1^xxiv^—As—N2^xxiv^	48.43 (5)
O4^xi^—N2—O3^xiii^	45.67 (7)	As^xxv^—O1—Ga2^xxvi^	137.99 (11)
O3^xii^—N2—O3^xiii^	88.14 (11)	As^xxv^—O1—N2^vi^	89.57 (11)
O3^v^—N2—O3^xiv^	150.86 (8)	Ga2^xxvi^—O1—N2^vi^	128.22 (11)
O3^iii^—N2—O3^xiv^	83.77 (6)	As^xxv^—O1—N2^xxv^	76.36 (10)
O3—N2—O3^xiv^	119.40 (8)	Ga2^xxvi^—O1—N2^xxv^	93.37 (8)
O1^vi^—N2—O3^xiv^	123.57 (16)	N2^vi^—O1—N2^xxv^	76.98 (4)
O1^vii^—N2—O3^xiv^	46.33 (6)	As^xxv^—O1—N2^xxvi^	121.95 (10)
O1^viii^—N2—O3^xiv^	63.78 (7)	Ga2^xxvi^—O1—N2^xxvi^	89.12 (8)
O4^ix^—N2—O3^xiv^	80.03 (12)	N2^vi^—O1—N2^xxvi^	74.93 (4)
O4^x^—N2—O3^xiv^	45.67 (7)	N2^xxv^—O1—N2^xxvi^	145.93 (11)
O4^xi^—N2—O3^xiv^	43.44 (7)	As—O2—Ga1^xxiv^	121.85 (9)
O3^xii^—N2—O3^xiv^	88.14 (11)	As—O2—N1	99.43 (7)
O3^xiii^—N2—O3^xiv^	88.14 (11)	Ga1^xxiv^—O2—N1	128.79 (7)
O2^xv^—Ga1—O2^iii^	91.61 (7)	As—O2—N2	60.17 (5)
O2^xv^—Ga1—O2^xvi^	91.61 (7)	Ga1^xxiv^—O2—N2	163.08 (8)
O2^iii^—Ga1—O2^xvi^	91.61 (7)	N1—O2—N2	63.03 (5)
O2^xv^—Ga1—O4^v^	88.91 (7)	As—O3—N2	129.17 (11)
O2^iii^—Ga1—O4^v^	92.29 (8)	As—O3—N1	105.03 (9)
O2^xvi^—Ga1—O4^v^	176.05 (7)	N2—O3—N1	93.77 (9)
O2^xv^—Ga1—O4^xvii^	92.29 (8)	As—O3—N2^xii^	82.43 (8)
O2^iii^—Ga1—O4^xvii^	176.05 (7)	N2—O3—N2^xii^	96.22 (6)
O2^xvi^—Ga1—O4^xvii^	88.91 (7)	N1—O3—N2^xii^	159.27 (9)
O4^v^—Ga1—O4^xvii^	87.16 (8)	As—O3—H3	102 (4)
O2^xv^—Ga1—O4^xviii^	176.05 (7)	N2—O3—H3	125 (4)
O2^iii^—Ga1—O4^xviii^	88.91 (7)	N1—O3—H3	91 (3)
O2^xvi^—Ga1—O4^xviii^	92.29 (7)	N2^xii^—O3—H3	69 (3)
O4^v^—Ga1—O4^xviii^	87.16 (8)	As^ii^—O4—Ga1^xxvi^	130.02 (10)
O4^xvii^—Ga1—O4^xviii^	87.16 (8)	As^ii^—O4—N2^xxvii^	85.25 (7)
O2^xv^—Ga1—N2^xxi^	124.12 (5)	Ga1^xxvi^—O4—N2^xxvii^	100.62 (8)
O2^iii^—Ga1—N2^xxi^	124.12 (5)	As^ii^—O4—N1^xxv^	124.11 (7)
O2^xvi^—Ga1—N2^xxi^	124.12 (5)	Ga1^xxvi^—O4—N1^xxv^	96.68 (6)
O4^v^—Ga1—N2^xxi^	52.75 (5)	N2^xxvii^—O4—N1^xxv^	118.40 (4)
O4^xvii^—Ga1—N2^xxi^	52.75 (5)	As^ii^—O4—N1	51.75 (5)
O4^xviii^—Ga1—N2^xxi^	52.75 (5)	Ga1^xxvi^—O4—N1	79.17 (5)
O2^xv^—Ga1—N1^xvii^	32.80 (5)	N2^xxvii^—O4—N1	104.63 (4)
O2^iii^—Ga1—N1^xvii^	105.75 (5)	N1^xxv^—O4—N1	136.70 (4)
O2^xvi^—Ga1—N1^xvii^	119.85 (5)	As^ii^—O4—N2^xxviii^	98.66 (7)
O4^v^—Ga1—N1^xvii^	59.54 (5)	Ga1^xxvi^—O4—N2^xxviii^	129.48 (6)
O4^xvii^—Ga1—N1^xvii^	77.33 (5)	N2^xxvii^—O4—N2^xxviii^	66.70 (3)
O4^xviii^—Ga1—N1^xvii^	143.46 (5)	N1^xxv^—O4—N2^xxviii^	57.01 (5)
N2^xxi^—Ga1—N1^xvii^	92.432 (5)	N1—O4—N2^xxviii^	150.37 (5)

Symmetry codes: (i) *x*−*y*, −*y*, −*z*+3/2; (ii) −*x*, −*x*+*y*, −*z*+3/2; (iii) −*x*+*y*, −*x*, *z*; (iv) *y*, *x*, −*z*+3/2; (v) −*y*, *x*−*y*, *z*; (vi) −*x*+2/3, −*y*−2/3, −*z*+4/3; (vii) *x*−*y*−4/3, *x*−2/3, −*z*+4/3; (viii) *y*+2/3, −*x*+*y*+4/3, −*z*+4/3; (ix) *x*−1/3, *x*−*y*−2/3, *z*−1/6; (x) −*y*−1/3, −*x*+1/3, *z*−1/6; (xi) −*x*+*y*+2/3, *y*+1/3, *z*−1/6; (xii) −*x*−1/3, −*y*−2/3, −*z*+4/3; (xiii) *y*+2/3, −*x*+*y*+1/3, −*z*+4/3; (xiv) *x*−*y*−1/3, *x*+1/3, −*z*+4/3; (xv) −*y*, *x*−*y*+1, *z*; (xvi) *x*+1, *y*+1, *z*; (xvii) *x*, *y*+1, *z*; (xviii) −*x*+*y*+1, −*x*+1, *z*; (xix) −*x*+2/3, −*y*+1/3, −*z*+4/3; (xx) *x*−1, *y*, *z*; (xxi) −*y*+1/3, −*x*+2/3, *z*+1/6; (xxii) −*x*−1/3, −*y*+1/3, −*z*+4/3; (xxiii) −*x*+2/3, −*y*+4/3, −*z*+4/3; (xxiv) *x*−1, *y*−1, *z*; (xxv) *x*+1, *y*, *z*; (xxvi) *x*, *y*−1, *z*; (xxvii) −*y*+1/3, −*x*−1/3, *z*+1/6; (xxviii) *y*+1, *x*, −*z*+3/2.

### Ammonium gallium bis[hydrogen arsenate(V)] (NH4GaHAsO42) Hydrogen-bond geometry (Å, º)

**Table:**

*D*—H···*A*	*D*—H	H···*A*	*D*···*A*	*D*—H···*A*
O3—H3···O4^xxix^	0.87 (3)	1.74 (3)	2.610 (3)	172 (6)

Symmetry code: (xxix) *y*, *x*−1, −*z*+3/2.

### Thallium aluminium bis[hydrogen arsenate(V)] (TlAlHAsO42) Crystal data

**Table:**

TlAl(HAsO_4_)_2_	*D*_x_ = 4.849 Mg m^−^^3^
*M**_r_* = 511.21	Mo *K*α radiation, λ = 0.71073 Å
Trigonal, *R*3*c*:*H*	Cell parameters from 1004 reflections
*a* = 8.290 (1) Å	θ = 2.9–30.0°
*c* = 52.940 (11) Å	µ = 32.58 mm^−^^1^
*V* = 3150.8 (10) Å^3^	*T* = 293 K
*Z* = 18	Small pseudo-octahedral platelets, colourless
*F*(000) = 4068	0.08 × 0.07 × 0.03 mm

### Thallium aluminium bis[hydrogen arsenate(V)] (TlAlHAsO42) Data collection

**Table:**

Nonius KappaCCD single-crystal four-circle diffractometer	685 reflections with *I* > 2σ(*I*)
Radiation source: fine-focus sealed tube	*R*_int_ = 0.022
φ and ω scans	θ_max_ = 26.0°, θ_min_ = 2.9°
Absorption correction: multi-scan (HKL SCALEPACK; Otwinowski *et al.*, 2003)	*h* = −10→10
*T*_min_ = 0.180, *T*_max_ = 0.441	*k* = −8→8
2478 measured reflections	*l* = −64→64
698 independent reflections	

### Thallium aluminium bis[hydrogen arsenate(V)] (TlAlHAsO42) Refinement

**Table:**

Refinement on *F*^2^	Secondary atom site location: difference Fourier map
Least-squares matrix: full	Hydrogen site location: difference Fourier map
*R*[*F*^2^ > 2σ(*F*^2^)] = 0.022	All H-atom parameters refined
*wR*(*F*^2^) = 0.058	*w* = 1/[σ^2^(*F*_o_^2^) + (0.023*P*)^2^ + 84.2452*P*] where *P* = (*F*_o_^2^ + 2*F*_c_^2^)/3
*S* = 1.21	(Δ/σ)_max_ = 0.003
698 reflections	Δρ_max_ = 0.82 e Å^−^^3^
69 parameters	Δρ_min_ = −1.98 e Å^−^^3^
2 restraints	Extinction correction: SHELXL2016 (Sheldrick, 2015), Fc^*^=kFc[1+0.001xFc^2^λ^3^/sin(2θ)]^-1/4^
Primary atom site location: structure-invariant direct methods	Extinction coefficient: 0.00049 (3)

### Thallium aluminium bis[hydrogen arsenate(V)] (TlAlHAsO42) Special details

**Table:**

Geometry. All esds (except the esd in the dihedral angle between two l.s. planes) are estimated using the full covariance matrix. The cell esds are taken into account individually in the estimation of esds in distances, angles and torsion angles; correlations between esds in cell parameters are only used when they are defined by crystal symmetry. An approximate (isotropic) treatment of cell esds is used for estimating esds involving l.s. planes.

### Thallium aluminium bis[hydrogen arsenate(V)] (TlAlHAsO42) Fractional atomic coordinates and isotropic or equivalent isotropic displacement parameters (Å^2^)

**Table:**

	*x*	*y*	*z*	*U*_iso_*/*U*_eq_	Occ. (<1)
Tl1	0.000000	−0.019 (2)	0.750000	0.037 (2)	0.3333
Tl2	0.000000	0.000000	0.66885 (2)	0.0322 (2)	
Al1	0.333333	0.666667	0.75439 (5)	0.0051 (5)	
Al2	0.333333	0.666667	0.666667	0.0061 (7)	
As	−0.43603 (7)	−0.39811 (7)	0.71289 (2)	0.00523 (19)	0.9790 (14)
AsB	−0.596 (3)	−0.559 (3)	0.7127 (4)	0.00523 (19)	0.0210 (14)
O1	0.4431 (5)	−0.4433 (5)	0.68625 (6)	0.0120 (8)	
O2	−0.4518 (5)	−0.2576 (5)	0.73421 (6)	0.0080 (7)	
O3	−0.2001 (5)	−0.2792 (5)	0.70491 (8)	0.0144 (8)	
O4	0.4791 (5)	−0.1259 (5)	0.77571 (6)	0.0083 (7)	
H3	−0.126 (8)	−0.323 (8)	0.7074 (11)	0.010 (15)*	

### Thallium aluminium bis[hydrogen arsenate(V)] (TlAlHAsO42) Atomic displacement parameters (Å^2^)

**Table:**

	*U*^11^	*U*^22^	*U*^33^	*U*^12^	*U*^13^	*U*^23^
Tl1	0.0356 (8)	0.051 (5)	0.0190 (4)	0.0178 (4)	−0.003 (2)	−0.0015 (10)
Tl2	0.0411 (3)	0.0411 (3)	0.0145 (3)	0.02053 (13)	0.000	0.000
Al1	0.0069 (7)	0.0069 (7)	0.0014 (11)	0.0035 (4)	0.000	0.000
Al2	0.0085 (11)	0.0085 (11)	0.0014 (16)	0.0042 (5)	0.000	0.000
As	0.0081 (3)	0.0070 (3)	0.0019 (3)	0.0047 (2)	0.00051 (17)	0.00064 (17)
AsB	0.0081 (3)	0.0070 (3)	0.0019 (3)	0.0047 (2)	0.00051 (17)	0.00064 (17)
O1	0.0188 (19)	0.0166 (18)	0.0032 (15)	0.0109 (16)	−0.0020 (14)	−0.0009 (13)
O2	0.0077 (17)	0.0103 (16)	0.0045 (15)	0.0034 (14)	0.0021 (13)	−0.0004 (13)
O3	0.0091 (18)	0.0168 (19)	0.0199 (19)	0.0085 (15)	0.0098 (15)	0.0102 (15)
O4	0.0119 (17)	0.0085 (17)	0.0049 (15)	0.0054 (14)	−0.0011 (13)	−0.0044 (13)

### Thallium aluminium bis[hydrogen arsenate(V)] (TlAlHAsO42) Geometric parameters (Å, º)

**Table:**

Tl1—Tl1^i^	0.28 (3)	Tl2—O3^xiv^	3.545 (4)
Tl1—Tl1^ii^	0.28 (3)	Tl2—O3^xv^	3.545 (4)
Tl1—O3	3.085 (8)	Tl2—AsB^xiii^	3.74 (2)
Tl1—O3^iii^	3.085 (8)	Tl2—AsB^xv^	3.74 (2)
Tl1—O3^iv^	3.136 (5)	Tl2—AsB^xiv^	3.74 (2)
Tl1—O3^i^	3.136 (5)	Al1—O2^xvi^	1.895 (4)
Tl1—O2^iv^	3.233 (13)	Al1—O2^ii^	1.895 (4)
Tl1—O2^i^	3.233 (13)	Al1—O2^xvii^	1.895 (4)
Tl1—O3^v^	3.261 (12)	Al1—O4^xviii^	1.901 (4)
Tl1—O3^ii^	3.261 (12)	Al1—O4^i^	1.901 (4)
Tl1—O2^iii^	3.351 (4)	Al1—O4^xix^	1.901 (4)
Tl1—O2	3.351 (4)	Al2—O1^ix^	1.887 (4)
Tl1—O2^ii^	3.501 (15)	Al2—O1^xv^	1.887 (4)
Tl1—O2^v^	3.501 (15)	Al2—O1^xx^	1.887 (4)
Tl1—AsB^vi^	3.89 (3)	Al2—O1^i^	1.887 (4)
Tl2—O3^i^	2.813 (4)	Al2—O1^xix^	1.887 (4)
Tl2—O3^ii^	2.813 (4)	Al2—O1^xviii^	1.887 (4)
Tl2—O3	2.813 (4)	As—AsB	1.33 (2)
Tl2—O1^vii^	3.410 (4)	As—O1^xxi^	1.661 (3)
Tl2—O1^viii^	3.410 (4)	As—O2	1.674 (3)
Tl2—O1^ix^	3.410 (4)	As—O4^iii^	1.679 (3)
Tl2—O4^x^	3.516 (3)	As—O3	1.746 (4)
Tl2—O4^xi^	3.516 (3)	AsB—O4^iii^	1.35 (2)
Tl2—O4^xii^	3.516 (3)	AsB—O1^xxi^	1.64 (2)
Tl2—O3^xiii^	3.545 (4)	AsB—O2^xxii^	2.12 (2)
			
Tl1^i^—Tl1—Tl1^ii^	60.00 (3)	O1^xix^—Al2—O1^xviii^	92.71 (15)
Tl1^i^—Tl1—O3	127.2 (3)	O1^ix^—Al2—Tl2^xx^	64.49 (12)
Tl1^ii^—Tl1—O3	98.0 (3)	O1^xv^—Al2—Tl2^xx^	64.45 (11)
Tl1^i^—Tl1—O3^iii^	98.0 (3)	O1^xx^—Al2—Tl2^xx^	145.30 (11)
Tl1^ii^—Tl1—O3^iii^	127.2 (2)	O1^i^—Al2—Tl2^xx^	115.51 (12)
O3—Tl1—O3^iii^	129.1 (6)	O1^xix^—Al2—Tl2^xx^	115.55 (11)
Tl1^i^—Tl1—O3^iv^	114.4 (3)	O1^xviii^—Al2—Tl2^xx^	34.70 (11)
Tl1^ii^—Tl1—O3^iv^	77.0 (4)	O1^ix^—Al2—Tl2^xviii^	115.51 (12)
O3—Tl1—O3^iv^	104.2 (3)	O1^xv^—Al2—Tl2^xviii^	115.55 (11)
O3^iii^—Tl1—O3^iv^	70.24 (16)	O1^xx^—Al2—Tl2^xviii^	34.70 (11)
Tl1^i^—Tl1—O3^i^	77.0 (3)	O1^i^—Al2—Tl2^xviii^	64.49 (12)
Tl1^ii^—Tl1—O3^i^	114.4 (2)	O1^xix^—Al2—Tl2^xviii^	64.45 (11)
O3—Tl1—O3^i^	70.24 (16)	O1^xviii^—Al2—Tl2^xviii^	145.29 (11)
O3^iii^—Tl1—O3^i^	104.2 (3)	Tl2^xx^—Al2—Tl2^xviii^	180.0
O3^iv^—Tl1—O3^i^	167.5 (6)	O1^ix^—Al2—Tl2^xvii^	115.55 (11)
Tl1^i^—Tl1—O2^iv^	163.77 (15)	O1^xv^—Al2—Tl2^xvii^	34.70 (11)
Tl1^ii^—Tl1—O2^iv^	112.8 (3)	O1^xx^—Al2—Tl2^xvii^	115.51 (12)
O3—Tl1—O2^iv^	66.4 (3)	O1^i^—Al2—Tl2^xvii^	64.45 (11)
O3^iii^—Tl1—O2^iv^	74.5 (3)	O1^xix^—Al2—Tl2^xvii^	145.29 (11)
O3^iv^—Tl1—O2^iv^	49.68 (16)	O1^xviii^—Al2—Tl2^xvii^	64.49 (12)
O3^i^—Tl1—O2^iv^	118.5 (5)	Tl2^xx^—Al2—Tl2^xvii^	60.1
Tl1^i^—Tl1—O2^i^	112.84 (18)	Tl2^xviii^—Al2—Tl2^xvii^	119.9
Tl1^ii^—Tl1—O2^i^	163.77 (10)	O1^ix^—Al2—Tl2	34.70 (11)
O3—Tl1—O2^i^	74.5 (3)	O1^xv^—Al2—Tl2	115.51 (12)
O3^iii^—Tl1—O2^i^	66.4 (3)	O1^xx^—Al2—Tl2	115.55 (12)
O3^iv^—Tl1—O2^i^	118.5 (5)	O1^i^—Al2—Tl2	145.29 (11)
O3^i^—Tl1—O2^i^	49.68 (16)	O1^xix^—Al2—Tl2	64.49 (12)
O2^iv^—Tl1—O2^i^	77.8 (4)	O1^xviii^—Al2—Tl2	64.45 (12)
Tl1^i^—Tl1—O3^v^	48.90 (18)	Tl2^xx^—Al2—Tl2	60.1
Tl1^ii^—Tl1—O3^v^	61.10 (14)	Tl2^xviii^—Al2—Tl2	119.942 (1)
O3—Tl1—O3^v^	158.5 (4)	Tl2^xvii^—Al2—Tl2	119.9
O3^iii^—Tl1—O3^v^	68.60 (12)	O1^ix^—Al2—Tl2^xxiv^	64.45 (11)
O3^iv^—Tl1—O3^v^	68.01 (16)	O1^xv^—Al2—Tl2^xxiv^	145.30 (11)
O3^i^—Tl1—O3^v^	121.2 (3)	O1^xx^—Al2—Tl2^xxiv^	64.49 (12)
O2^iv^—Tl1—O3^v^	115.03 (10)	O1^i^—Al2—Tl2^xxiv^	115.55 (11)
O2^i^—Tl1—O3^v^	127.04 (11)	O1^xix^—Al2—Tl2^xxiv^	34.70 (11)
Tl1^i^—Tl1—O3^ii^	61.1 (2)	O1^xviii^—Al2—Tl2^xxiv^	115.51 (12)
Tl1^ii^—Tl1—O3^ii^	48.90 (16)	Tl2^xx^—Al2—Tl2^xxiv^	119.9
O3—Tl1—O3^ii^	68.60 (12)	Tl2^xviii^—Al2—Tl2^xxiv^	60.1
O3^iii^—Tl1—O3^ii^	158.5 (4)	Tl2^xvii^—Al2—Tl2^xxiv^	180.0
O3^iv^—Tl1—O3^ii^	121.2 (3)	Tl2—Al2—Tl2^xxiv^	60.1
O3^i^—Tl1—O3^ii^	68.01 (16)	O1^ix^—Al2—Tl2^xxv^	145.30 (11)
O2^iv^—Tl1—O3^ii^	127.04 (11)	O1^xv^—Al2—Tl2^xxv^	64.49 (12)
O2^i^—Tl1—O3^ii^	115.03 (10)	O1^xx^—Al2—Tl2^xxv^	64.45 (12)
O3^v^—Tl1—O3^ii^	97.6 (5)	O1^i^—Al2—Tl2^xxv^	34.70 (11)
Tl1^i^—Tl1—O2^iii^	62.8 (3)	O1^xix^—Al2—Tl2^xxv^	115.51 (12)
Tl1^ii^—Tl1—O2^iii^	120.68 (19)	O1^xviii^—Al2—Tl2^xxv^	115.55 (12)
O3—Tl1—O2^iii^	129.1 (3)	Tl2^xx^—Al2—Tl2^xxv^	119.9
O3^iii^—Tl1—O2^iii^	48.95 (11)	Tl2^xviii^—Al2—Tl2^xxv^	60.1
O3^iv^—Tl1—O2^iii^	115.18 (11)	Tl2^xvii^—Al2—Tl2^xxv^	60.1
O3^i^—Tl1—O2^iii^	64.40 (9)	Tl2—Al2—Tl2^xxv^	180.0
O2^iv^—Tl1—O2^iii^	117.7 (4)	Tl2^xxiv^—Al2—Tl2^xxv^	119.9
O2^i^—Tl1—O2^iii^	59.15 (18)	AsB—As—O1^xxi^	65.3 (9)
O3^v^—Tl1—O2^iii^	70.66 (15)	AsB—As—O2	108.5 (9)
O3^ii^—Tl1—O2^iii^	111.8 (3)	O1^xxi^—As—O2	118.77 (18)
Tl1^i^—Tl1—O2	120.7 (3)	AsB—As—O4^iii^	51.8 (9)
Tl1^ii^—Tl1—O2	62.8 (4)	O1^xxi^—As—O4^iii^	106.18 (18)
O3—Tl1—O2	48.94 (10)	O2—As—O4^iii^	114.71 (17)
O3^iii^—Tl1—O2	129.1 (3)	AsB—As—O3	147.0 (10)
O3^iv^—Tl1—O2	64.40 (9)	O1^xxi^—As—O3	107.52 (19)
O3^i^—Tl1—O2	115.18 (11)	O2—As—O3	103.03 (19)
O2^iv^—Tl1—O2	59.15 (17)	O4^iii^—As—O3	105.63 (17)
O2^i^—Tl1—O2	117.7 (4)	AsB—As—Tl2^xiii^	79.0 (9)
O3^v^—Tl1—O2	111.8 (3)	O1^xxi^—As—Tl2^xiii^	65.02 (13)
O3^ii^—Tl1—O2	70.66 (15)	O2—As—Tl2^xiii^	172.42 (13)
O2^iii^—Tl1—O2	176.5 (6)	O4^iii^—As—Tl2^xiii^	68.63 (12)
Tl1^i^—Tl1—O2^ii^	14.95 (6)	O3—As—Tl2^xiii^	69.39 (14)
Tl1^ii^—Tl1—O2^ii^	55.40 (8)	AsB—As—Tl1^ii^	150.1 (9)
O3—Tl1—O2^ii^	112.4 (2)	O1^xxi^—As—Tl1^ii^	142.74 (15)
O3^iii^—Tl1—O2^ii^	112.3 (2)	O2—As—Tl1^ii^	54.3 (2)
O3^iv^—Tl1—O2^ii^	122.2 (4)	O4^iii^—As—Tl1^ii^	109.18 (17)
O3^i^—Tl1—O2^ii^	70.1 (2)	O3—As—Tl1^ii^	51.5 (2)
O2^iv^—Tl1—O2^ii^	168.2 (3)	Tl2^xiii^—As—Tl1^ii^	118.4 (2)
O2^i^—Tl1—O2^ii^	113.58 (8)	AsB—As—Tl1	149.5 (9)
O3^v^—Tl1—O2^ii^	61.5 (3)	O1^xxi^—As—Tl1	144.6 (2)
O3^ii^—Tl1—O2^ii^	46.5 (2)	O2—As—Tl1	57.8 (2)
O2^iii^—Tl1—O2^ii^	72.6 (2)	O4^iii^—As—Tl1	106.1 (2)
O2—Tl1—O2^ii^	110.7 (4)	O3—As—Tl1	49.19 (16)
Tl1^i^—Tl1—O2^v^	55.40 (16)	Tl2^xiii^—As—Tl1	115.07 (16)
Tl1^ii^—Tl1—O2^v^	14.95 (6)	Tl1^ii^—As—Tl1	4.1 (4)
O3—Tl1—O2^v^	112.3 (2)	AsB—As—Tl1^i^	151.5 (9)
O3^iii^—Tl1—O2^v^	112.4 (2)	O1^xxi^—As—Tl1^i^	142.12 (17)
O3^iv^—Tl1—O2^v^	70.1 (2)	O2—As—Tl1^i^	56.77 (13)
O3^i^—Tl1—O2^v^	122.2 (4)	O4^iii^—As—Tl1^i^	108.88 (15)
O2^iv^—Tl1—O2^v^	113.58 (7)	O3—As—Tl1^i^	49.06 (16)
O2^i^—Tl1—O2^v^	168.2 (3)	Tl2^xiii^—As—Tl1^i^	115.97 (5)
O3^v^—Tl1—O2^v^	46.5 (2)	Tl1^ii^—As—Tl1^i^	2.5 (3)
O3^ii^—Tl1—O2^v^	61.5 (3)	Tl1—As—Tl1^i^	2.8 (3)
O2^iii^—Tl1—O2^v^	110.7 (4)	AsB—As—Tl2	145.5 (9)
O2—Tl1—O2^v^	72.6 (2)	O1^xxi^—As—Tl2	83.71 (13)
O2^ii^—Tl1—O2^v^	55.3 (3)	O2—As—Tl2	99.45 (13)
Tl1^i^—Tl1—AsB^vi^	135.2 (4)	O4^iii^—As—Tl2	131.69 (12)
Tl1^ii^—Tl1—AsB^vi^	141.1 (4)	O3—As—Tl2	30.29 (12)
O3—Tl1—AsB^vi^	43.1 (4)	Tl2^xiii^—As—Tl2	74.034 (11)
O3^iii^—Tl1—AsB^vi^	89.5 (5)	Tl1^ii^—As—Tl2	64.11 (8)
O3^iv^—Tl1—AsB^vi^	109.6 (5)	Tl1—As—Tl2	63.95 (6)
O3^i^—Tl1—AsB^vi^	58.5 (4)	Tl1^i^—As—Tl2	62.34 (12)
O2^iv^—Tl1—AsB^vi^	60.1 (4)	AsB—As—Tl1^xxvi^	23.3 (9)
O2^i^—Tl1—AsB^vi^	33.0 (4)	O1^xxi^—As—Tl1^xxvi^	87.98 (15)
O3^v^—Tl1—AsB^vi^	157.7 (4)	O2—As—Tl1^xxvi^	93.41 (14)
O3^ii^—Tl1—AsB^vi^	101.9 (3)	O4^iii^—As—Tl1^xxvi^	41.90 (12)
O2^iii^—Tl1—AsB^vi^	92.0 (4)	O3—As—Tl1^xxvi^	147.50 (13)
O2—Tl1—AsB^vi^	85.0 (4)	Tl2^xiii^—As—Tl1^xxvi^	93.30 (5)
O2^ii^—Tl1—AsB^vi^	127.8 (3)	Tl1^ii^—As—Tl1^xxvi^	126.79 (12)
O2^v^—Tl1—AsB^vi^	155.4 (3)	Tl1—As—Tl1^xxvi^	126.30 (17)
O3^i^—Tl2—O3^ii^	79.02 (13)	Tl1^i^—As—Tl1^xxvi^	128.27 (4)
O3^i^—Tl2—O3	79.02 (13)	Tl2—As—Tl1^xxvi^	166.91 (6)
O3^ii^—Tl2—O3	79.02 (13)	AsB—As—Tl1^xxii^	22.1 (9)
O3^i^—Tl2—O1^vii^	75.43 (10)	O1^xxi^—As—Tl1^xxii^	86.49 (17)
O3^ii^—Tl2—O1^vii^	154.16 (10)	O2—As—Tl1^xxii^	93.06 (16)
O3—Tl2—O1^vii^	92.26 (10)	O4^iii^—As—Tl1^xxii^	43.7 (2)
O3^i^—Tl2—O1^viii^	154.16 (10)	O3—As—Tl1^xxii^	149.3 (2)
O3^ii^—Tl2—O1^viii^	92.26 (10)	Tl2^xiii^—As—Tl1^xxii^	93.75 (10)
O3—Tl2—O1^viii^	75.43 (10)	Tl1^ii^—As—Tl1^xxii^	127.913 (17)
O1^vii^—Tl2—O1^viii^	109.19 (6)	Tl1—As—Tl1^xxii^	127.56 (3)
O3^i^—Tl2—O1^ix^	92.26 (10)	Tl1^i^—As—Tl1^xxii^	129.46 (17)
O3^ii^—Tl2—O1^ix^	75.43 (10)	Tl2—As—Tl1^xxii^	166.76 (4)
O3—Tl2—O1^ix^	154.15 (10)	Tl1^xxvi^—As—Tl1^xxii^	1.9 (2)
O1^vii^—Tl2—O1^ix^	109.19 (6)	As—AsB—O4^iii^	77.6 (12)
O1^viii^—Tl2—O1^ix^	109.19 (6)	As—AsB—O1^xxi^	67.1 (10)
O3^i^—Tl2—O4^x^	110.02 (10)	O4^iii^—AsB—O1^xxi^	126.4 (16)
O3^ii^—Tl2—O4^x^	153.52 (10)	As—AsB—O2^xxii^	112.5 (13)
O3—Tl2—O4^x^	126.54 (10)	O4^iii^—AsB—O2^xxii^	119.3 (13)
O1^vii^—Tl2—O4^x^	45.33 (8)	O1^xxi^—AsB—O2^xxii^	111.1 (11)
O1^viii^—Tl2—O4^x^	88.61 (8)	As—AsB—Tl2^xiii^	80.6 (10)
O1^ix^—Tl2—O4^x^	79.30 (8)	O4^iii^—AsB—Tl2^xiii^	69.9 (9)
O3^i^—Tl2—O4^xi^	153.52 (10)	O1^xxi^—AsB—Tl2^xiii^	65.6 (7)
O3^ii^—Tl2—O4^xi^	126.54 (10)	O2^xxii^—AsB—Tl2^xiii^	164.8 (9)
O3—Tl2—O4^xi^	110.02 (11)	As—AsB—Tl1^xxvi^	149.0 (12)
O1^vii^—Tl2—O4^xi^	79.30 (8)	O4^iii^—AsB—Tl1^xxvi^	84.2 (10)
O1^viii^—Tl2—O4^xi^	45.33 (8)	O1^xxi^—AsB—Tl1^xxvi^	142.5 (11)
O1^ix^—Tl2—O4^xi^	88.61 (8)	O2^xxii^—AsB—Tl1^xxvi^	56.2 (5)
O4^x^—Tl2—O4^xi^	44.26 (9)	Tl2^xiii^—AsB—Tl1^xxvi^	116.5 (6)
O3^i^—Tl2—O4^xii^	126.54 (11)	As—AsB—Tl1^xxii^	150.8 (12)
O3^ii^—Tl2—O4^xii^	110.01 (10)	O4^iii^—AsB—Tl1^xxii^	86.9 (10)
O3—Tl2—O4^xii^	153.52 (11)	O1^xxi^—AsB—Tl1^xxii^	140.0 (11)
O1^vii^—Tl2—O4^xii^	88.61 (8)	O2^xxii^—AsB—Tl1^xxii^	54.8 (5)
O1^viii^—Tl2—O4^xii^	79.30 (8)	Tl2^xiii^—AsB—Tl1^xxii^	117.2 (6)
O1^ix^—Tl2—O4^xii^	45.33 (8)	Tl1^xxvi^—AsB—Tl1^xxii^	2.7 (3)
O4^x^—Tl2—O4^xii^	44.26 (9)	As—AsB—Tl1^xxvii^	150.4 (12)
O4^xi^—Tl2—O4^xii^	44.26 (9)	O4^iii^—AsB—Tl1^xxvii^	83.6 (10)
O3^i^—Tl2—O3^xiii^	117.91 (14)	O1^xxi^—AsB—Tl1^xxvii^	141.8 (11)
O3^ii^—Tl2—O3^xiii^	152.25 (13)	O2^xxii^—AsB—Tl1^xxvii^	58.4 (6)
O3—Tl2—O3^xiii^	82.85 (11)	Tl2^xiii^—AsB—Tl1^xxvii^	114.1 (6)
O1^vii^—Tl2—O3^xiii^	46.50 (9)	Tl1^xxvi^—AsB—Tl1^xxvii^	2.4 (3)
O1^viii^—Tl2—O3^xiii^	62.74 (9)	Tl1^xxii^—AsB—Tl1^xxvii^	3.9 (4)
O1^ix^—Tl2—O3^xiii^	122.31 (9)	As—AsB—Tl2^xxvii^	146.6 (12)
O4^x^—Tl2—O3^xiii^	45.47 (8)	O4^iii^—AsB—Tl2^xxvii^	112.1 (11)
O4^xi^—Tl2—O3^xiii^	42.94 (8)	O1^xxi^—AsB—Tl2^xxvii^	82.8 (8)
O4^xii^—Tl2—O3^xiii^	78.63 (8)	O2^xxii^—AsB—Tl2^xxvii^	91.1 (7)
O3^i^—Tl2—O3^xiv^	82.85 (11)	Tl2^xiii^—AsB—Tl2^xxvii^	73.8 (4)
O3^ii^—Tl2—O3^xiv^	117.91 (14)	Tl1^xxvi^—AsB—Tl2^xxvii^	64.0 (4)
O3—Tl2—O3^xiv^	152.25 (12)	Tl1^xxii^—AsB—Tl2^xxvii^	62.3 (3)
O1^vii^—Tl2—O3^xiv^	62.74 (9)	Tl1^xxvii^—AsB—Tl2^xxvii^	62.2 (3)
O1^viii^—Tl2—O3^xiv^	122.31 (9)	As—AsB—Tl1^ii^	22.5 (7)
O1^ix^—Tl2—O3^xiv^	46.50 (9)	O4^iii^—AsB—Tl1^ii^	66.6 (9)
O4^x^—Tl2—O3^xiv^	42.94 (9)	O1^xxi^—AsB—Tl1^ii^	88.7 (8)
O4^xi^—Tl2—O3^xiv^	78.63 (8)	O2^xxii^—AsB—Tl1^ii^	99.8 (7)
O4^xii^—Tl2—O3^xiv^	45.47 (8)	Tl2^xiii^—AsB—Tl1^ii^	95.0 (5)
O3^xiii^—Tl2—O3^xiv^	87.33 (9)	Tl1^xxvi^—AsB—Tl1^ii^	126.5 (5)
O3^i^—Tl2—O3^xv^	152.25 (13)	Tl1^xxii^—AsB—Tl1^ii^	128.3 (5)
O3^ii^—Tl2—O3^xv^	82.85 (11)	Tl1^xxvii^—AsB—Tl1^ii^	128.0 (5)
O3—Tl2—O3^xv^	117.91 (14)	Tl2^xxvii^—AsB—Tl1^ii^	168.1 (5)
O1^vii^—Tl2—O3^xv^	122.31 (9)	As—AsB—Tl1	22.9 (7)
O1^viii^—Tl2—O3^xv^	46.50 (9)	O4^iii^—AsB—Tl1	63.7 (9)
O1^ix^—Tl2—O3^xv^	62.74 (9)	O1^xxi^—AsB—Tl1	89.8 (9)
O4^x^—Tl2—O3^xv^	78.63 (8)	O2^xxii^—AsB—Tl1	102.1 (7)
O4^xi^—Tl2—O3^xv^	45.47 (8)	Tl2^xiii^—AsB—Tl1	92.8 (5)
O4^xii^—Tl2—O3^xv^	42.94 (8)	Tl1^xxvi^—AsB—Tl1	126.1 (5)
O3^xiii^—Tl2—O3^xv^	87.33 (9)	Tl1^xxii^—AsB—Tl1	128.1 (5)
O3^xiv^—Tl2—O3^xv^	87.33 (9)	Tl1^xxvii^—AsB—Tl1	127.5 (5)
O3^i^—Tl2—AsB^xiii^	90.6 (3)	Tl2^xxvii^—AsB—Tl1	166.5 (6)
O3^ii^—Tl2—AsB^xiii^	159.8 (4)	Tl1^ii^—AsB—Tl1	3.1 (3)
O3—Tl2—AsB^xiii^	116.2 (4)	As—AsB—Tl2	26.4 (7)
O1^vii^—Tl2—AsB^xiii^	25.9 (3)	O4^iii^—AsB—Tl2	87.6 (10)
O1^viii^—Tl2—AsB^xiii^	104.1 (4)	O1^xxi^—AsB—Tl2	44.9 (7)
O1^ix^—Tl2—AsB^xiii^	87.9 (4)	O2^xxii^—AsB—Tl2	128.4 (8)
O4^x^—Tl2—AsB^xiii^	21.2 (3)	Tl2^xiii^—AsB—Tl2	60.8 (3)
O4^xi^—Tl2—AsB^xiii^	63.0 (3)	Tl1^xxvi^—AsB—Tl2	171.8 (6)
O4^xii^—Tl2—AsB^xiii^	62.7 (3)	Tl1^xxii^—AsB—Tl2	174.5 (6)
O3^xiii^—Tl2—AsB^xiii^	47.7 (4)	Tl1^xxvii^—AsB—Tl2	171.0 (6)
O3^xiv^—Tl2—AsB^xiii^	42.9 (4)	Tl2^xxvii^—AsB—Tl2	120.2 (4)
O3^xv^—Tl2—AsB^xiii^	99.8 (3)	Tl1^ii^—AsB—Tl2	48.6 (2)
O3^i^—Tl2—AsB^xv^	159.8 (4)	Tl1—AsB—Tl2	48.5 (2)
O3^ii^—Tl2—AsB^xv^	116.2 (4)	AsB^xxviii^—O1—As^xxviii^	47.6 (8)
O3—Tl2—AsB^xv^	90.6 (4)	AsB^xxviii^—O1—Al2^xxix^	138.8 (8)
O1^vii^—Tl2—AsB^xv^	87.9 (4)	As^xxviii^—O1—Al2^xxix^	137.7 (2)
O1^viii^—Tl2—AsB^xv^	25.9 (3)	AsB^xxviii^—O1—Tl2^vii^	88.4 (8)
O1^ix^—Tl2—AsB^xv^	104.1 (3)	As^xxviii^—O1—Tl2^vii^	88.78 (15)
O4^x^—Tl2—AsB^xv^	62.7 (3)	Al2^xxix^—O1—Tl2^vii^	126.91 (15)
O4^xi^—Tl2—AsB^xv^	21.2 (3)	AsB^xxviii^—O1—Tl2^xxix^	75.1 (8)
O4^xii^—Tl2—AsB^xv^	63.0 (3)	As^xxviii^—O1—Tl2^xxix^	121.09 (16)
O3^xiii^—Tl2—AsB^xv^	42.9 (4)	Al2^xxix^—O1—Tl2^xxix^	92.35 (12)
O3^xiv^—Tl2—AsB^xv^	99.8 (3)	Tl2^vii^—O1—Tl2^xxix^	75.55 (7)
O3^xv^—Tl2—AsB^xv^	47.7 (4)	AsB^xxviii^—O1—Tl2^xxviii^	119.6 (8)
AsB^xiii^—Tl2—AsB^xv^	78.5 (5)	As^xxviii^—O1—Tl2^xxviii^	73.85 (13)
O3^i^—Tl2—AsB^xiv^	116.2 (4)	Al2^xxix^—O1—Tl2^xxviii^	92.31 (12)
O3^ii^—Tl2—AsB^xiv^	90.6 (4)	Tl2^vii^—O1—Tl2^xxviii^	75.53 (7)
O3—Tl2—AsB^xiv^	159.8 (4)	Tl2^xxix^—O1—Tl2^xxviii^	146.93 (9)
O1^vii^—Tl2—AsB^xiv^	104.1 (4)	As—O2—Al1^xxvii^	122.5 (2)
O1^viii^—Tl2—AsB^xiv^	87.9 (4)	As—O2—AsB^xxx^	104.8 (6)
O1^ix^—Tl2—AsB^xiv^	25.9 (3)	Al1^xxvii^—O2—AsB^xxx^	102.5 (6)
O4^x^—Tl2—AsB^xiv^	63.0 (3)	As—O2—Tl1^ii^	100.8 (2)
O4^xi^—Tl2—AsB^xiv^	62.7 (3)	Al1^xxvii^—O2—Tl1^ii^	128.4 (2)
O4^xii^—Tl2—AsB^xiv^	21.2 (3)	AsB^xxx^—O2—Tl1^ii^	90.8 (7)
O3^xiii^—Tl2—AsB^xiv^	99.8 (3)	As—O2—Tl1	97.2 (3)
O3^xiv^—Tl2—AsB^xiv^	47.7 (4)	Al1^xxvii^—O2—Tl1	130.04 (17)
O3^xv^—Tl2—AsB^xiv^	42.9 (4)	AsB^xxx^—O2—Tl1	94.2 (7)
AsB^xiii^—Tl2—AsB^xiv^	78.5 (5)	Tl1^ii^—O2—Tl1	4.4 (5)
AsB^xv^—Tl2—AsB^xiv^	78.5 (5)	As—O2—Tl1^i^	99.66 (14)
O2^xvi^—Al1—O2^ii^	91.34 (17)	Al1^xxvii^—O2—Tl1^i^	129.68 (16)
O2^xvi^—Al1—O2^xvii^	91.34 (17)	AsB^xxx^—O2—Tl1^i^	90.6 (7)
O2^ii^—Al1—O2^xvii^	91.34 (17)	Tl1^ii^—O2—Tl1^i^	1.27 (13)
O2^xvi^—Al1—O4^xviii^	92.20 (15)	Tl1—O2—Tl1^i^	3.9 (4)
O2^ii^—Al1—O4^xviii^	176.43 (17)	As—O2—Tl2	60.21 (10)
O2^xvii^—Al1—O4^xviii^	88.17 (15)	Al1^xxvii^—O2—Tl2	163.54 (15)
O2^xvi^—Al1—O4^i^	88.17 (15)	AsB^xxx^—O2—Tl2	62.4 (6)
O2^ii^—Al1—O4^i^	92.20 (16)	Tl1^ii^—O2—Tl2	61.76 (9)
O2^xvii^—Al1—O4^i^	176.43 (18)	Tl1—O2—Tl2	61.25 (6)
O4^xviii^—Al1—O4^i^	88.32 (17)	Tl1^i^—O2—Tl2	60.58 (8)
O2^xvi^—Al1—O4^xix^	176.43 (17)	As—O3—Tl2	131.46 (18)
O2^ii^—Al1—O4^xix^	88.16 (15)	As—O3—Tl1	105.45 (17)
O2^xvii^—Al1—O4^xix^	92.20 (15)	Tl2—O3—Tl1	93.5 (2)
O4^xviii^—Al1—O4^xix^	88.32 (17)	As—O3—Tl1^ii^	102.7 (3)
O4^i^—Al1—O4^xix^	88.32 (17)	Tl2—O3—Tl1^ii^	92.38 (13)
O2^xvi^—Al1—Tl2^xxiii^	124.31 (12)	Tl1—O3—Tl1^ii^	5.0 (5)
O2^ii^—Al1—Tl2^xxiii^	124.31 (12)	As—O3—Tl1^i^	107.1 (3)
O2^xvii^—Al1—Tl2^xxiii^	124.31 (12)	Tl2—O3—Tl1^i^	89.8 (3)
O4^xviii^—Al1—Tl2^xxiii^	53.56 (12)	Tl1—O3—Tl1^i^	3.9 (4)
O4^i^—Al1—Tl2^xxiii^	53.56 (12)	Tl1^ii^—O3—Tl1^i^	4.5 (5)
O4^xix^—Al1—Tl2^xxiii^	53.56 (12)	As—O3—Tl2^xiii^	83.17 (14)
O2^xvi^—Al1—Tl1^xviii^	32.98 (11)	Tl2—O3—Tl2^xiii^	97.14 (11)
O2^ii^—Al1—Tl1^xviii^	104.20 (15)	Tl1—O3—Tl2^xiii^	156.2 (3)
O2^xvii^—Al1—Tl1^xviii^	120.62 (14)	Tl1^ii^—O3—Tl2^xiii^	161.0 (3)
O4^xviii^—Al1—Tl1^xviii^	79.07 (14)	Tl1^i^—O3—Tl2^xiii^	158.83 (12)
O4^i^—Al1—Tl1^xviii^	57.99 (12)	AsB^iii^—O4—As^iii^	50.7 (10)
O4^xix^—Al1—Tl1^xviii^	143.97 (14)	AsB^iii^—O4—Al1^xxix^	170.4 (10)
Tl2^xxiii^—Al1—Tl1^xviii^	92.86 (3)	As^iii^—O4—Al1^xxix^	130.5 (2)
O2^xvi^—Al1—Tl1^i^	120.61 (14)	AsB^iii^—O4—Tl2^xxxi^	88.9 (9)
O2^ii^—Al1—Tl1^i^	32.98 (11)	As^iii^—O4—Tl2^xxxi^	84.96 (12)
O2^xvii^—Al1—Tl1^i^	104.20 (15)	Al1^xxix^—O4—Tl2^xxxi^	100.65 (14)
O4^xviii^—Al1—Tl1^i^	143.97 (14)	AsB^iii^—O4—Tl1^xxxii^	76.1 (10)
O4^i^—Al1—Tl1^i^	79.07 (14)	As^iii^—O4—Tl1^xxxii^	121.76 (16)
O4^xix^—Al1—Tl1^i^	57.99 (13)	Al1^xxix^—O4—Tl1^xxxii^	98.16 (12)
Tl2^xxiii^—Al1—Tl1^i^	92.86 (3)	Tl2^xxxi^—O4—Tl1^xxxii^	119.60 (14)
Tl1^xviii^—Al1—Tl1^i^	119.753 (11)	AsB^iii^—O4—Tl1^xxviii^	77.7 (10)
O2^xvi^—Al1—Tl1^xix^	104.20 (16)	As^iii^—O4—Tl1^xxviii^	124.1 (3)
O2^ii^—Al1—Tl1^xix^	120.61 (14)	Al1^xxix^—O4—Tl1^xxviii^	96.88 (19)
O2^xvii^—Al1—Tl1^xix^	32.98 (12)	Tl2^xxxi^—O4—Tl1^xxviii^	117.56 (15)
O4^xviii^—Al1—Tl1^xix^	57.99 (13)	Tl1^xxxii^—O4—Tl1^xxviii^	2.8 (3)
O4^i^—Al1—Tl1^xix^	143.97 (14)	AsB^iii^—O4—Tl1^xxxiii^	74.5 (10)
O4^xix^—Al1—Tl1^xix^	79.07 (15)	As^iii^—O4—Tl1^xxxiii^	120.5 (2)
Tl2^xxiii^—Al1—Tl1^xix^	92.86 (4)	Al1^xxix^—O4—Tl1^xxxiii^	99.8 (2)
Tl1^xviii^—Al1—Tl1^xix^	119.753 (7)	Tl2^xxxi^—O4—Tl1^xxxiii^	118.95 (9)
Tl1^i^—Al1—Tl1^xix^	119.753 (6)	Tl1^xxxii^—O4—Tl1^xxxiii^	1.61 (17)
O2^xvi^—Al1—Tl1^xvi^	32.35 (12)	Tl1^xxviii^—O4—Tl1^xxxiii^	3.7 (4)
O2^ii^—Al1—Tl1^xvi^	106.6 (2)	AsB^iii^—O4—Tl1	101.3 (10)
O2^xvii^—Al1—Tl1^xvi^	118.5 (2)	As^iii^—O4—Tl1	53.74 (18)
O4^xviii^—Al1—Tl1^xvi^	76.73 (19)	Al1^xxix^—O4—Tl1	77.44 (19)
O4^i^—Al1—Tl1^xvi^	59.94 (18)	Tl2^xxxi^—O4—Tl1	103.75 (9)
O4^xix^—Al1—Tl1^xvi^	144.77 (15)	Tl1^xxxii^—O4—Tl1	136.34 (9)
Tl2^xxiii^—Al1—Tl1^xvi^	92.78 (3)	Tl1^xxviii^—O4—Tl1	138.6 (2)
Tl1^xviii^—Al1—Tl1^xvi^	2.9 (3)	Tl1^xxxiii^—O4—Tl1	136.76 (8)
Tl1^i^—Al1—Tl1^xvi^	122.7 (3)	AsB^iii^—O4—Tl1^i^	98.2 (10)
Tl1^xix^—Al1—Tl1^xvi^	116.9 (3)	As^iii^—O4—Tl1^i^	51.25 (16)
O2^xvi^—Al1—Tl1^ii^	118.5 (2)	Al1^xxix^—O4—Tl1^i^	80.3 (2)
O2^ii^—Al1—Tl1^ii^	32.35 (12)	Tl2^xxxi^—O4—Tl1^i^	105.06 (14)
O2^xvii^—Al1—Tl1^ii^	106.6 (2)	Tl1^xxxii^—O4—Tl1^i^	134.6 (3)
O4^xviii^—Al1—Tl1^ii^	144.77 (15)	Tl1^xxviii^—O4—Tl1^i^	136.94 (8)
O4^i^—Al1—Tl1^ii^	76.7 (2)	Tl1^xxxiii^—O4—Tl1^i^	134.9 (2)
O4^xix^—Al1—Tl1^ii^	59.94 (19)	Tl1—O4—Tl1^i^	3.3 (4)
Tl2^xxiii^—Al1—Tl1^ii^	92.78 (3)	AsB^iii^—O4—Tl2^xxxiv^	53.0 (10)
Tl1^xviii^—Al1—Tl1^ii^	116.9 (3)	As^iii^—O4—Tl2^xxxiv^	97.70 (13)
Tl1^i^—Al1—Tl1^ii^	2.9 (3)	Al1^xxix^—O4—Tl2^xxxiv^	130.13 (14)
Tl1^xix^—Al1—Tl1^ii^	122.7 (3)	Tl2^xxxi^—O4—Tl2^xxxiv^	67.31 (5)
Tl1^xvi^—Al1—Tl1^ii^	119.767 (7)	Tl1^xxxii^—O4—Tl2^xxxiv^	56.91 (9)
O1^ix^—Al2—O1^xv^	92.72 (15)	Tl1^xxviii^—O4—Tl2^xxxiv^	55.99 (5)
O1^ix^—Al2—O1^xx^	92.72 (15)	Tl1^xxxiii^—O4—Tl2^xxxiv^	55.69 (6)
O1^xv^—Al2—O1^xx^	92.72 (15)	Tl1—O4—Tl2^xxxiv^	151.34 (16)
O1^ix^—Al2—O1^i^	180.0	Tl1^i^—O4—Tl2^xxxiv^	148.95 (14)
O1^xv^—Al2—O1^i^	87.28 (15)	AsB^iii^—O4—Tl1^ii^	99.9 (10)
O1^xx^—Al2—O1^i^	87.28 (15)	As^iii^—O4—Tl1^ii^	52.32 (10)
O1^ix^—Al2—O1^xix^	87.28 (15)	Al1^xxix^—O4—Tl1^ii^	78.86 (11)
O1^xv^—Al2—O1^xix^	180.0	Tl2^xxxi^—O4—Tl1^ii^	103.45 (10)
O1^xx^—Al2—O1^xix^	87.28 (15)	Tl1^xxxii^—O4—Tl1^ii^	136.45 (9)
O1^i^—Al2—O1^xix^	92.71 (15)	Tl1^xxviii^—O4—Tl1^ii^	138.7 (2)
O1^ix^—Al2—O1^xviii^	87.28 (15)	Tl1^xxxiii^—O4—Tl1^ii^	136.81 (8)
O1^xv^—Al2—O1^xviii^	87.28 (15)	Tl1—O4—Tl1^ii^	1.42 (15)
O1^xx^—Al2—O1^xviii^	180.00 (17)	Tl1^i^—O4—Tl1^ii^	2.3 (3)
O1^i^—Al2—O1^xviii^	92.71 (15)	Tl2^xxxiv^—O4—Tl1^ii^	149.95 (8)

Symmetry codes: (i) −*y*, *x*−*y*, *z*; (ii) −*x*+*y*, −*x*, *z*; (iii) −*x*, −*x*+*y*, −*z*+3/2; (iv) *y*, *x*, −*z*+3/2; (v) *x*−*y*, −*y*, −*z*+3/2; (vi) −*x*+*y*, −*x*−1, *z*; (vii) −*x*+2/3, −*y*−2/3, −*z*+4/3; (viii) *x*−*y*−4/3, *x*−2/3, −*z*+4/3; (ix) *y*+2/3, −*x*+*y*+4/3, −*z*+4/3; (x) *x*−1/3, *x*−*y*−2/3, *z*−1/6; (xi) −*y*−1/3, −*x*+1/3, *z*−1/6; (xii) −*x*+*y*+2/3, *y*+1/3, *z*−1/6; (xiii) −*x*−1/3, −*y*−2/3, −*z*+4/3; (xiv) *y*+2/3, −*x*+*y*+1/3, −*z*+4/3; (xv) *x*−*y*−1/3, *x*+1/3, −*z*+4/3; (xvi) −*y*, *x*−*y*+1, *z*; (xvii) *x*+1, *y*+1, *z*; (xviii) *x*, *y*+1, *z*; (xix) −*x*+*y*+1, −*x*+1, *z*; (xx) −*x*+2/3, −*y*+1/3, −*z*+4/3; (xxi) *x*−1, *y*, *z*; (xxii) −*x*+*y*−1, −*x*−1, *z*; (xxiii) −*y*+1/3, −*x*+2/3, *z*+1/6; (xxiv) −*x*−1/3, −*y*+1/3, −*z*+4/3; (xxv) −*x*+2/3, −*y*+4/3, −*z*+4/3; (xxvi) −*y*−1, *x*−*y*−1, *z*; (xxvii) *x*−1, *y*−1, *z*; (xxviii) *x*+1, *y*, *z*; (xxix) *x*, *y*−1, *z*; (xxx) −*y*−1, *x*−*y*, *z*; (xxxi) −*y*+1/3, −*x*−1/3, *z*+1/6; (xxxii) −*x*+*y*+1, −*x*, *z*; (xxxiii) −*y*+1, *x*−*y*, *z*; (xxxiv) *y*+1, *x*, −*z*+3/2.

### Thallium aluminium bis[hydrogen arsenate(V)] (TlAlHAsO42) Hydrogen-bond geometry (Å, º)

**Table:**

*D*—H···*A*	*D*—H	H···*A*	*D*···*A*	*D*—H···*A*
O3—H3···O4^xxxv^	0.87 (4)	1.87 (5)	2.584 (5)	139 (6)

Symmetry code: (xxxv) *y*, *x*−1, −*z*+3/2.
